# Genome-wide mapping of main histone modifications and coordination regulation of metabolic genes under salt stress in pea (*Pisum sativum L*)

**DOI:** 10.1093/hr/uhae259

**Published:** 2024-09-16

**Authors:** Heping Wan, Lan Cao, Ping Wang, Hanbing Hu, Rui Guo, Jingdong Chen, Huixia Zhao, Changli Zeng, Xiaoyun Liu

**Affiliations:** Hubei Engineering Research Center for Protection and Utilization of Special Biological Resources in the Hanjiang River Basin, College of Life Sciences, Jianghan University, Sanjiaohu Road, Wuhan Economic and Technological Development Zone, Hubei 430056, China; Hubei Engineering Research Center for Protection and Utilization of Special Biological Resources in the Hanjiang River Basin, College of Life Sciences, Jianghan University, Sanjiaohu Road, Wuhan Economic and Technological Development Zone, Hubei 430056, China; Hubei Engineering Research Center for Protection and Utilization of Special Biological Resources in the Hanjiang River Basin, College of Life Sciences, Jianghan University, Sanjiaohu Road, Wuhan Economic and Technological Development Zone, Hubei 430056, China; Hubei Engineering Research Center for Protection and Utilization of Special Biological Resources in the Hanjiang River Basin, College of Life Sciences, Jianghan University, Sanjiaohu Road, Wuhan Economic and Technological Development Zone, Hubei 430056, China; Hubei Engineering Research Center for Protection and Utilization of Special Biological Resources in the Hanjiang River Basin, College of Life Sciences, Jianghan University, Sanjiaohu Road, Wuhan Economic and Technological Development Zone, Hubei 430056, China; Hubei Engineering Research Center for Protection and Utilization of Special Biological Resources in the Hanjiang River Basin, College of Life Sciences, Jianghan University, Sanjiaohu Road, Wuhan Economic and Technological Development Zone, Hubei 430056, China; Hubei Engineering Research Center for Protection and Utilization of Special Biological Resources in the Hanjiang River Basin, College of Life Sciences, Jianghan University, Sanjiaohu Road, Wuhan Economic and Technological Development Zone, Hubei 430056, China

## Abstract

Pea occupy a key position in modern biogenetics, playing multifaceted roles as food, vegetable, fodder, and green manure. However, due to the complex nature of its genome and the prolonged unveiling of high-quality genetic maps, research into the molecular mechanisms underlying pea development and stress responses has been significantly delayed. Furthermore, the exploration of its epigenetic modification profiles and associated regulatory mechanisms remains uncharted. This research conducted a comprehensive investigation of four specific histone marks, namely H3K4me3, H3K27me3, H3K9ac, and H3K9me2, and the transcriptome in pea under normal conditions, and established a global map of genome-wide regulatory elements, chromatin states, and dynamics based on these major modifications. Our analysis identified epigenomic signals across ~82.6% of the genome. Each modification exhibits distinct enrichment patterns: H3K4me3 is predominantly associated with the gibberellin response pathway, H3K27me3 is primarily associated with auxin and ethylene responses, and H3K9ac is primarily associated with negative regulatory stimulus responses. We also identified a novel bivalent chromatin state (H3K9ac-H3K27me3) in pea, which is related to their development and stress response. Additionally, we unveil that these histone modifications synergistically regulate metabolic-related genes, influencing metabolite production under salt stress conditions. Our findings offer a panoramic view of the major histone modifications in pea, elucidate their interplay, and highlight their transcriptional regulatory roles during salt stress.

## Introduction

Soil salinization stands as a predominant abiotic stress in agricultural production, increasingly impeding crop growth and yield [1]. Current data suggests that nearly 20% of cropland and 50% of irrigated areas worldwide are adversely affected by soil salinity. This escalating problem poses a significant challenge to the sustainable progression of global agriculture [2].

Salt-induced damage pertains to the adverse effects of elevated salt concentrations on plant growth, encompassing both osmotic stress and ionic toxicity [3]. Elevated soil salt concentration significantly reduces soil water potential, thereby promoting water loss from the soil [1]. Under such saline conditions, plants experience an excessive accumulation of Na^+^ ions in their tissues and cells, leading to ion toxicity [4, 5]. Concurrently, salt stress conditions prompt an increased production of reactive oxygen species (ROS) in plants, including superoxide radicals (·O_2_^−^), hydroxyl free radicals (·OH), and hydrogen peroxide (H_2_O_2_). Excessive intracellular ROS cause damage to DNA structures, resulting in mutations within key functional genes. These mutations impair gene expression, which is vital for sustaining essential cellular physiological processes, and can ultimately trigger cellular senescence and death [6]. In adaptation to these adversities, plants have evolved a myriad of adaptive strategies, notably the synthesis of osmotic regulators, ROS detoxification, and ion homeostasis [7].

Histone methylation and histone acetylation, as two prevalent modifications among histone modifications, serve as pivotal epigenetic markers in the regulation of gene expression. Research has elucidated that H3K4me3 and H3K9ac modifications are predominantly located near the transcription start site (TSS) of genes and are associated with the activation of gene transcription. Conversely, H3K27me3 modification is typically enriched within the gene body and correlates with transcriptional repression [8]. Notably, H3K4me3 and H3K27me3 are frequently perceived as antagonistic markers, modulating the expression of target genes in reaction to environmental fluctuations during plant growth and maturation [9, 10]. Recent research has underscored the involvement of histone modifications in plant responses to salt stress [11]. For instance, in *Arabidopsis*, JMJ15 bolsters salt stress resilience by suppressing the WKRY transcription factor through H3K4me3 removal under salt stress conditions [12]. Under saline and alkaline stresses, the rice protein SDG721, possessing H3K4 methyltransferase activity, binds to both the promoter and coding regions of HKT1, depositing H3K4 markers to modulate its expression [13]. The H3K27me3 marker is profusely enriched in *Arabidopsis* HKT1’s gene body, with HKT1 activation under salt stress resulting from H3K27me3 removal [14]. In soybean plants, the suppression of numerous genes during salt stress is tightly associated with the new formation of H3K27me3 across different promoter regions or coding sequences [15]. A comparative analysis between the salt-tolerant rice varieties Nonabokra and IR64 reveals that under salt stress, there are significant differences in chromatin modifications (H3K4me3 and H3K27me3) between rice varieties regulating the differential expression of the OsBZ8 gene [16]. Beyond histone methylation’s role in salt stress, salt-induced histone acetylation has been documented to be intrinsically linked to the transcriptional activation of salt stress-associated genes across diverse species [17–23]. In *Arabidopsis*, Class I histone deacetylases (HDA6, HDA9, HDA19) enhance positive adaptations to salt stress, whereas Class II RPD3 histone deacetylases (HDA5, HDA14, HDA15, HDA18) contribute to adverse responses [22, 24, 25]. In *Hibiscus cannabinus*, several histone deacetylases and acetylation levels at H3K9ac, H3K27ac, and H4K5ac have been observed to elevate under salt stress conditions [26]. GCN5, in *Arabidopsis*, augments gene transcription levels under salt stress, modulating salt tolerance. It binds to target genes, such as CTL1, PGX3, and MYB54, adjusting their expression in response to salt stress by influencing H3K9ac and H3K14ac levels [27]. SGF29, another component of the GCN5 complex, has been identified in mutants exhibiting salt stress resistance [28]. In soybean plants subjected to priming salinity treatment, histone marks like H3K4me2, H3K4me3, and H3K9ac were notably induced, collectively influencing stress response, cell wall adjustments, and ion balance [29]. Interestingly, the inhibition of histone acetylation in non-primed plants triggered transcriptional responses similar to those observed in primed plants, highlighting the critical importance of histone modifications in priming effects [29]. These findings collectively demonstrate the pivotal role of both histone methylation and acetylation in regulating the expression of genes responsive to salt stress, emphasizing their essential function in the epigenetic regulation of salt stress adaptation.

Pea (*Pisum sativum* L., 2n = 2x = 14) is a multipurpose crop, distinguishable into grain pea and vegetative pea, the latter recognized for their tender pods [30]. Additionally, pea are utilized as fodder and green manure, highlighting their multifunctional roles in agriculture [31]. Relative to soybeans and broad beans, pea exhibit heightened sensitivity to salinity [32]. Saline conditions significantly impact pea germination rates, as well as fresh and dry weights, water levels, seedling vigor, and salt tolerance index, demonstrating the adverse effects of salinity on pea growth and development [33]. Such stress can also lead to significant yield reductions, with studies noting nearly a 50% yield decrease at a 100 mM NaCl concentration [34]. However, the molecular mechanisms by which pea plants respond to salt stress remain largely unclear.

Historically, pea has been instrumental in advancing plant genetics, serving as a classic model of genetic research [35, 36]. Despite their significance, the complex nature of the pea genome has historically hindered the development of high-quality genome maps. This limitation has stymied genetic analyses of key agronomic traits in pea and hampered the exploration and utilization of germplasm resources and associated theoretical research. Fortunately, with the advent of advanced sequencing technologies, a high-quality pea genome map was realized in 2022. This achievement promises to usher in a new era of accelerated functional genome research in pea [30].

Epigenetic modifications play a pivotal role in gene transcription regulation, which not only directly impact an organism’s energy metabolism [37] but also affect numerous biological functions such as plant growth, resistance to stress, and determinants of yield and quality. While significant progress has been made in epigenetic research in model plants like *Arabidopsis* and rice [38, 39], with various chromatin modifications having been elucidated [39, 40], our understanding of the pea genome remains nascent. Current epigenetic research of pea is predominantly confined to DNA methylation [41, 42], leaving vast areas uncharted. Furthermore, analogous to the profound influence of an animal’s diet on its individual epigenetic modifications [43], the cultivation environment of plants also exerts a substantial effect on their epigenetic landscape [11]. Notably, the epigenetic regulatory mechanisms underlying pea’s response to stress conditions remain elusive.

In this study, we employed chromatin immunoprecipitation sequencing (ChIP-seq) to systematically profile four classic histone modifications (H3K4me3, H3K27me3, H3K9ac, and H3K9me2) in pea seedlings. This endeavor marks the first comprehensive analysis characterizing the distribution, organization, and gene expression relationships of these modifications within the pea genome. We established a global map of genome-wide regulatory elements, chromatin states, and dynamics based on these major modifications. Approximately 82.6% of the genome was marked by diverse epigenomic signals. And each modification has its own enrichment characteristics. We also discovered a new bivalent chromatin state (H3K9ac-H3K27me3) in pea, which is related to their development and stress response. Integrating transcriptomics and metabolomics, we further explored the influences of these histone modifications on metabolite accumulation, particularly in relation to Phenylpropanoid biosynthesis metabolism post-salt stress. Collectively, our study offers a comprehensive understanding of how histone modifications, gene transcription, and metabolic changes interact in reaction to salt stress in peas.

## Results

### Characterization of histone modifications in pea

**Figure 1:**
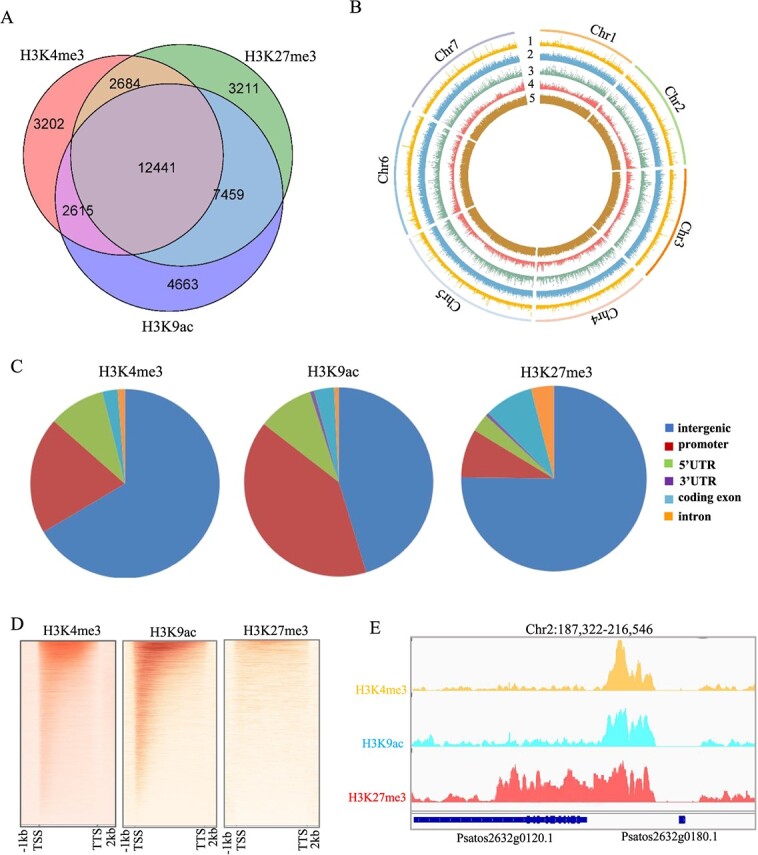
**Histone modification profiles in pea seedlings analyzed by ChIP-Seq. A.** The number of H3K4me3-, H3K27me3-, and H3K9ac-marked genes in pea seedlings. **B.** Circos plots displaying the distribution of H3K4me3, H3K27me3, and H3K9ac across the seven chromosomes of the pea genome. Numbered tracks: 1, H3K27me3 modification levels; 2, H3K4me3 modification levels; 3, H3K9ac modification levels; 4, genes; 5, repeat. **C.** The distribution of histone modifications within different regions of the pea genome. **D.** Distribution of the three modifications relative to gene positions in pea, showing a metaplot of normalized H3K4me3, H3K27me3, and H3K9ac across protein-coding genes from −1 kb upstream to +2 kb downstream of the TSS and TTS. **E.** Genome browser screenshot showing ChIP-Seq data example.

To elucidate the epigenomic landscape in pea, we generated genome-wide distribution of four predominant histone marks (H3K4me3, H3K9ac, H3K27me3, and H3K9me2) in pea seedlings, complemented by transcriptome data. We assessed the quality of the data by examining metrics such as the total read count, alignment rate, and data reproducibility (Table S1, Fig. S1). Furthermore, we corroborated the ChIP-seq and RNA-seq findings (Fig. S1) through quantitative Real-time PCR (RT-qPCR) assays on three randomly selected genes. At the same time, 10 genes enriched with H3K4me3, H3K9ac, and H3K27me3 modifications, respectively, were selected from the data for RT-qPCR validation, further verifying the accuracy of the ChIP-seq data (Fig. S2). After filtering out low-quality peaks and focusing on the three hallmark histone modifications, our ChIP-seq analysis yielded 55 681 (H3K4me3), 51 175 (H3K9ac), 56 868 (H3K27me3) peaks, and 75 337 (H3K9me2), spanning roughly 13.6%, 16.60%, 13.7%, and 54.1% of the genome, respectively (Table S2). The regions marked by H3K4me3, H3K9ac, H3K27me3, and H3K9me2 encompass 20 942, 27 178, 25 759, and 12 018 genes, which constitute ~46.8%, 60.7%, 57.6%, and 26.8% of the entire gene set, respectively (Table S2). Since H3K9me2 modification is mainly enriched in heterochromatin regions (Fig. S3), subsequent gene-related analysis mainly focuses on the other three modifications. Previous calculations suggest that nearly 80% of genes exhibit enrichment in at least one of these three histone modifications, with H3K9ac modification being more prevalent in the pea genome relative to the other two (Table S2, Fig. 1A). Moreover, we discerned genes marked by multiple modifications, including 15 125 (H3K4me3/H3K27me3), 15 056 (H3K4me3/H3K9ac), 19 900 (H3K4me3/H3K9ac), and 12 441 (H3K4me3/H3K9ac/H3K27me3) (Fig. 1A). These findings indicate that the majority of pea genes are likely regulated by one or more types of histone modifications.

Regarding the pea chromosome structure, a significant portion of the seven chromosomes is dominated by repetitive sequences [30, 44]. Interestingly, we observed that H3K4me3, H3K9ac, and H3K27me3 are dispersed across both repetitive sequences and genes in pea (Fig. 1B). This distribution contrasts with other species where these modifications are typically confined to the euchromatin domain [9, 44–48]. The predominant lengths of regions marked by these three histone modifications ranged from 200 to 700 bp (Fig. S4).

To clarify how gene distribution patterns relate to histone modifications, genomic elements were classified into six specific categories: five associated with gene regions and one linked to intergenic regions. Our research findings indicate that these three modifications are most abundantly distributed in promoter regions, excluding intergenic regions. Specifically, H3K9ac exhibits the highest proportion of distribution on promoters compared to the other two modifications. Both H3K4me3 and H3K9ac are also relatively more prevalent in the 5’ UTR region. Conversely, H3K27me3 is more abundantly distributed in coding exons and introns than the other two modifications (Fig. 1C). Aligning with prior research in other organisms [9, 44, 46, 47], we discerned that H3K4me3 and H3K9ac are primarily situated around the TSS of genes (exhibiting significant co-localization), while H3K27me3 are more dispersed throughout the genome (Fig. 1D–E).

Gene Ontology (GO) analysis of genes affected by H3K4me3, H3K9ac, and H3K27me3 revealed that genes with H3K4me3 modifications are predominantly involved in meristem development (encompassing shoot apex, floral development, and root growth) and gibberellin responses. H3K27me3-marked genes are chiefly associated with tissue-specific differentiation, floral and organ development, floral morphogenesis, and responses to auxin and ethylene. Meanwhile, H3K9ac-marked genes are primarily linked to stimulus response regulation, tissue differentiation, floral and organ development, and floral morphogenesis (Fig. S5). These observations underscore the pivotal roles of all three modifications in modulating plant organogenesis and growth. While H3K4me3 and H3K27me3 exhibit specific predilections in hormone response regulation—with H3K4me3 favoring gibberellin and H3K27me3 leaning toward ethylene and auxin—the H3K9ac enrichment is more attuned to stimulus responses.

Our dataset indicates that over half of the genes are marked by two or more histone modifications (Fig. 1A). Consequently, we also examined the modification distribution in genes marked by multiple histone modifications. The data indicates that the modification distribution of genes co-marked by H3K4me3 and H3K9ac is relatively even, with no bias toward either H3K4me3 modification or H3K9ac modification. However, genes co-marked by H3K27me3 and H3K4me3 lean more toward H3K27me3, as do genes marked by H3K27me3 and H3K9ac (Fig. S6). While H3K4me3 and H3K27me3 are recognized as bivalent modifications in numerous species, our findings hint at the possibility of H3K27me3 and H3K9ac emerging as a novel bivalent modification. This hypothesis, however, warrants further experimental validation. The distribution patterns of genes marked by these three histone modifications align closely with H3K27me3 (Fig. S7A). Collectively, these insights offer a comprehensive perspective on the primary histone modification landscape in pea.

### Analysis of the correlation between histone modification and mRNA transcription

**Figure 2:**
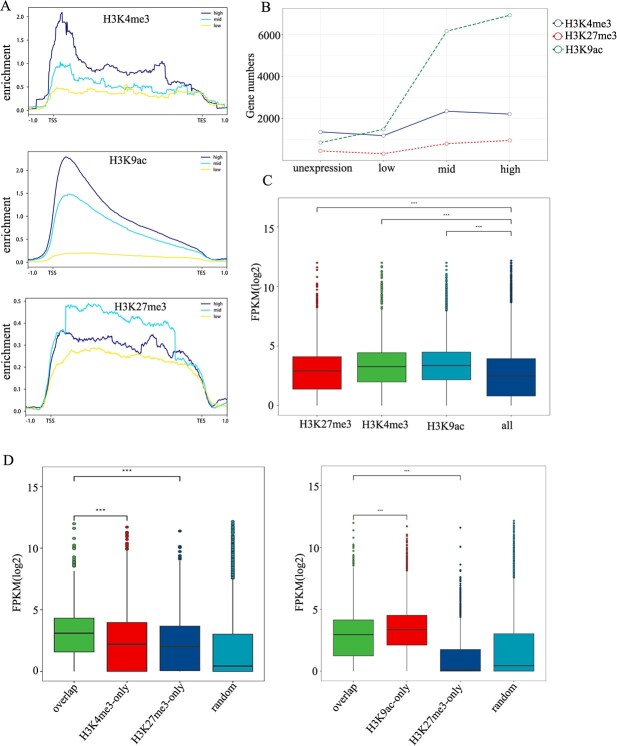
**Correlation of histone marks with mRNA expression levels. A.** Genes categorized by expression levels: high (FPKM >10), mid (FPKM >1 and FPKM <10), low (FPKM <1), showing distribution variations of methylation or acetylation depth (H3K4me3, H3K27me3, H3K9ac). **B.** Changes in the number of genes marked by H3K4me3, H3K27me3, or H3K9ac among different expression level groups. **C.** Average expression levels of genes marked by H3K4me3, H3K27me3, and H3K9ac compared to all genes in control pea seedlings, with significant differences indicated by asterisks (Wilcoxon test, *P* < 0.001). **D.** Expression breadth of genes in indicated categories, with statistical significance tested using the Wilcoxon rank-sum test; ‘random’ refers to 10 000 randomly selected genes.

To elucidate the interplay between the three histone modifications and gene expression in pea, and to discern the influence of each individual modification on transcriptional activity, we undertook a joint analysis of ChIP-seq and transcriptome data from pea seedlings. We enumerated genes marked by H3K4me3, H3K9ac, and H3K27me3 and categorized them based on their expression levels. Genes were stratified into: high (FPKM >10), medium (1 < FPKM <10), low (FPKM <1), and non-expressed (FPKM = 0) categories. Subsequent analysis of the distribution of these three modifications across these categories revealed that genes marked with H3K4me3 and H3K9ac were predominantly enriched among the highly expressed genes. Conversely, genes characterized by the presence of H3K27me3 were predominantly linked to genes exhibiting medium levels of expression (Fig. 2A). This deviates from prior research that typically associates H3K27me3 with lowly expressed genes. Additionally, our comparative analysis of the gene counts marked by the three histone modifications revealed a pronounced positive correlation between H3K9ac- and H3K4me3-marked genes. H3K27me3, on the other hand, displayed a relatively consistent gene count across different expression levels, albeit with a slight preponderance toward highly expressed genes (Fig. 2B). Additionally, genes marked by H3K9ac and H3K4me3 displayed significantly elevated expression levels than the average expression levels of all genes (Wilcox. test, *P* < 0.001). Even genes marked by H3K27me3 demonstrated marginally enhanced expression levels (Wilcox. test, *P* < 0.001) (Fig. 2C).

In our subsequent analysis, we explored the expression patterns of genes marked by single versus multiple histone modifications. The findings indicated that genes co-marked by H3K4me3 and H3K27me3 exhibited higher expression levels than those with singly modification marks. Meanwhile, genes co-marked by H3K9ac and H3K27me3 displayed expression levels intermediate between the two singly marked gene sets, leaning more toward the expression levels of H3K9ac-marked genes (Fig. 2D). Remarkably, genes co-marked by all three modifications mirrored the expression patterns of H3K9ac-marked genes but exhibited a significant uptick in expression levels compared to genes marked by the other two modifications. These results suggest that the presence of H3K9ac may play a dominant role in driving the expression of these co-regulated genes, resulting in enhanced expression levels (Fig. S7B–C).

### Chromatin states, genome function, and transcription in pea

**Figure 3:**
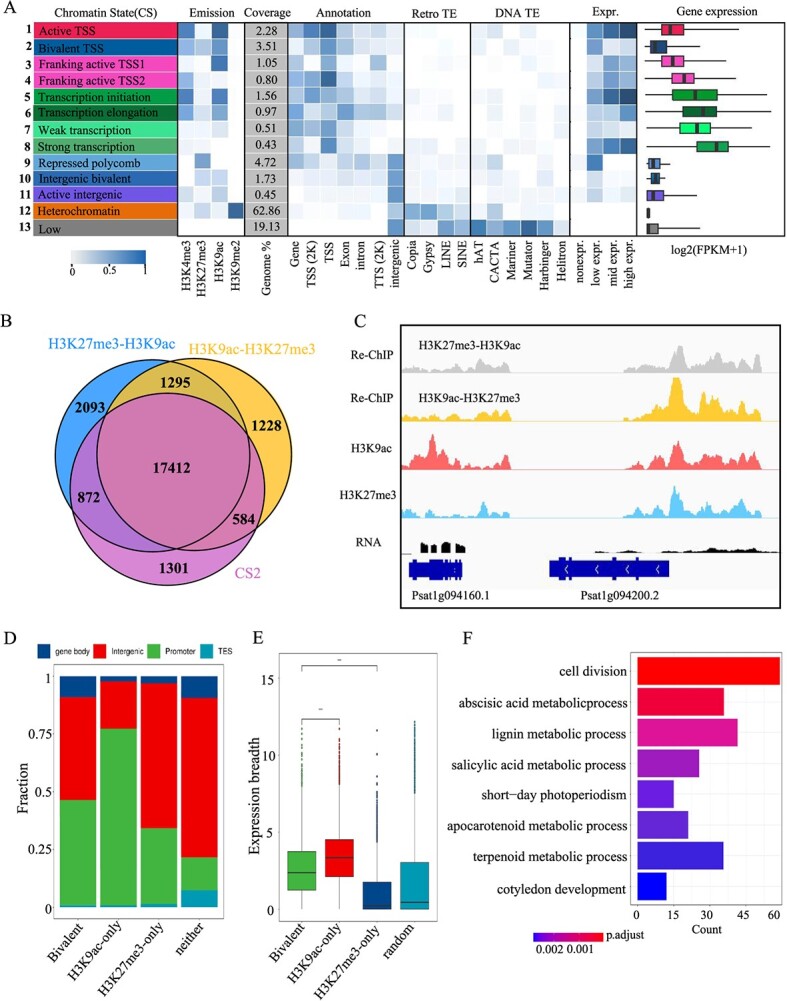
**Chromatin states and bivalent chromatin state characterization in pea. A.** Definitions, abbreviations, and composition (emission probability) of histone marks in chromatin states. **B.** Venn diagram showing overlap of targets enriched with H3K27me3-H3K9ac-reChIP and H3K9ac-H3K27me3-reChIP within CS 2 regions. **C.** Genome browser snapshot of H3K27me3-H3K9ac-reChIP and H3K9ac-H3K27me3-reChIP data for a representative gene in a CS 11 region**. D.** Distribution of regions with bivalent modifications, H3K9ac, and H3K27me3 in the pea genome; ‘neither’ refers to regions lacking examined epigenetic marks**. E.** Expression breadth of genes in specified categories; the Wilcoxon rank-sum test was used for statistical significance testing; ‘random’ refers to 10 000 randomly selected genes. **F.** GO enrichment of genes marked with bivalent modifications; the color scale represents the significance (*P*-adjust) of enriched GO terms.

Subsequently, we employed ChromHMM22 [49] to systematically delineate chromatin states by integrating the intricate patterns of three histone marks (Fig. 3A), identifying a 13-chromatin states (CS) model. We observed that active states comprised an average of 13.3% of the reference epigenome, primarily including active promoter-related states (CS1), transcription-related states (CS3–8), and active intergenic states (CS11). The repressed states (CS2, 9, 10, 12) and no signal (CS1,3) each exhibited transposable element (TE) enrichment, distinct genomic coverage, and levels of gene expression (Fig. 3A). The active promoters and transcription-related states typically exhibited high frequencies of H3K4me3 and H3K9ac and low frequencies of H3K27me3, covering 7.6% of the genome. The intergenic active state (CS11) was primarily associated with H3K4me3 and H3K9ac, encompassing most candidate enhancers or other regions proximal to expressed genes.

We observed that repressed states encompassed ~9.96% of the pea genome, including classical bivalent promoter states (CS2), bivalent intergenic states (CS10), and ReprPC (CS9). The bivalent promoter state, besides the known bivalent states of H3K4me3 and H3K27me3, exhibited a unique bivalent state of H3K9ac and H3K27me3, accounting for ~3.51% of the genome. This novel bivalent state was also present in intergenic regions, with the bivalent H3K9ac and H3K27me3 states accounting for ~1.73% of the genome. CS Repressed polycomb was primarily marked by H3K27me3 alone and was primarily enriched in intergenic regions, where it was associated with higher levels of DNA transposons (Fig. 3A).

The heterochromatin state (CS12), characterized by the enrichment of H3K9me2, spanned 62.86% of the pea genome. Zones linked to CS12 displayed the greatest concentration of TEs and the lowest levels of transcription. Predominantly, these areas overlapped with TEs, especially retrotransposons like Gypsy, Copia, and LINE, as well as DNA transposons. In contrast, the low state (CS13), which showed none of the epigenetic markers under study, encompassed annotated intergenic areas of the genome. This state was notably rich in LINE and SINE retrotransposons and DNA transposons (Fig. 3A).

In summary, these data reveal a map of different genomic regions and highlight the complex relationships between histone modifications, TE enrichment, and gene expression across different CSs. Apart from the low state, >80% of the pea genome was characterized by at least one CS.

### A new bivalent chromatin state correlated with H3K27me3 and H3K9ac in pea

Our epigenomic mapping of pea has identified a novel bivalent CS characterized by co-occurrence of H3K9ac and H3K27me3 modifications (bivalent gene states/CS2 and CS10), distinct from the classical H3K4me3-H3K27me3 bivalent state. Typically, H3K27me3 is associated with repressive chromatin, while H3K9ac is found at TSSs in plants. This bivalent CS encompasses 31.08% of H3K27me3 peaks and 24.34% of H3K9ac peaks, covering 15 921 genes. The authenticity of CS2 and CS10 was validated utilizing ChIP-reChIP assays on young pea leaf tissues. The analyses indicated that 86.33% of the newly identified bivalent CS regions were overlapping, establishing the presence of a bona fide H3K9ac-H3K27me3 bivalent CS in the pea genome (Fig. 3B–C). Additionally, RT-qPCR analysis was conducted on 10 genes with H3K9ac-H3K27me3 bivalent modifications, and the results closely matched the sequencing data, affirming the data’s accuracy (Fig. S8). This bivalent modification is predominantly found in gene promoter areas, aligning with the locations of H3K9ac and H3K27me3 (Fig. 3D). Additionally, we observed that genes marked by the novel bivalent modification exhibit a high degree of tissue-specific expression patterns, positioned between genes marked by H3K9ac and H3K27me3 (Fig. 3E), implying a possible regulatory function of the H3K9ac-H3K27me3 bivalent CS in specific gene expression within pea. GO enrichment of genes marked by the bivalent CS and found that these genes revealed their involvement in processes such as cell division, short-day photoperiodism, ABA, and SA metabolic processes, as well as secondary metabolite processes such as lignin, apocarotenoid, and terpenoid metabolism (Fig. 3F). These findings indicate that the novel bivalent CS may be crucial in pea development, growth, and stress response.

### Morphological and physiological alterations in leaves following salinity stress

To understand the role of histone modification in the stress response of pea, we treated pea with salt stress. Upon subjecting pea seeds to a 1.0% (m/v) NaCl salt treatment from germination through to the seedling growth phase for 20 days, pronounced phenotypic variations were evident. These included diminished leaf size and a shift to a pale-yellow hue (Fig. S9A–B). Relative to the control group, salt-treated seedlings exhibited a marked reduction in both shoot fresh weight (SFW) and root fresh weight (RFW), coupled with a significant decrease in average leaf area. These observations underscore the inhibitory effect of salt stress on pea seedling growth and development. Specifically, salt stress curtailed the accumulation of shoot and root biomass and diminished the concentration of vitamin C, soluble sugars, and soluble proteins in leaf tissues.

### Dynamics of histone modification and gene expression post-salt treatment

**Figure 4:**
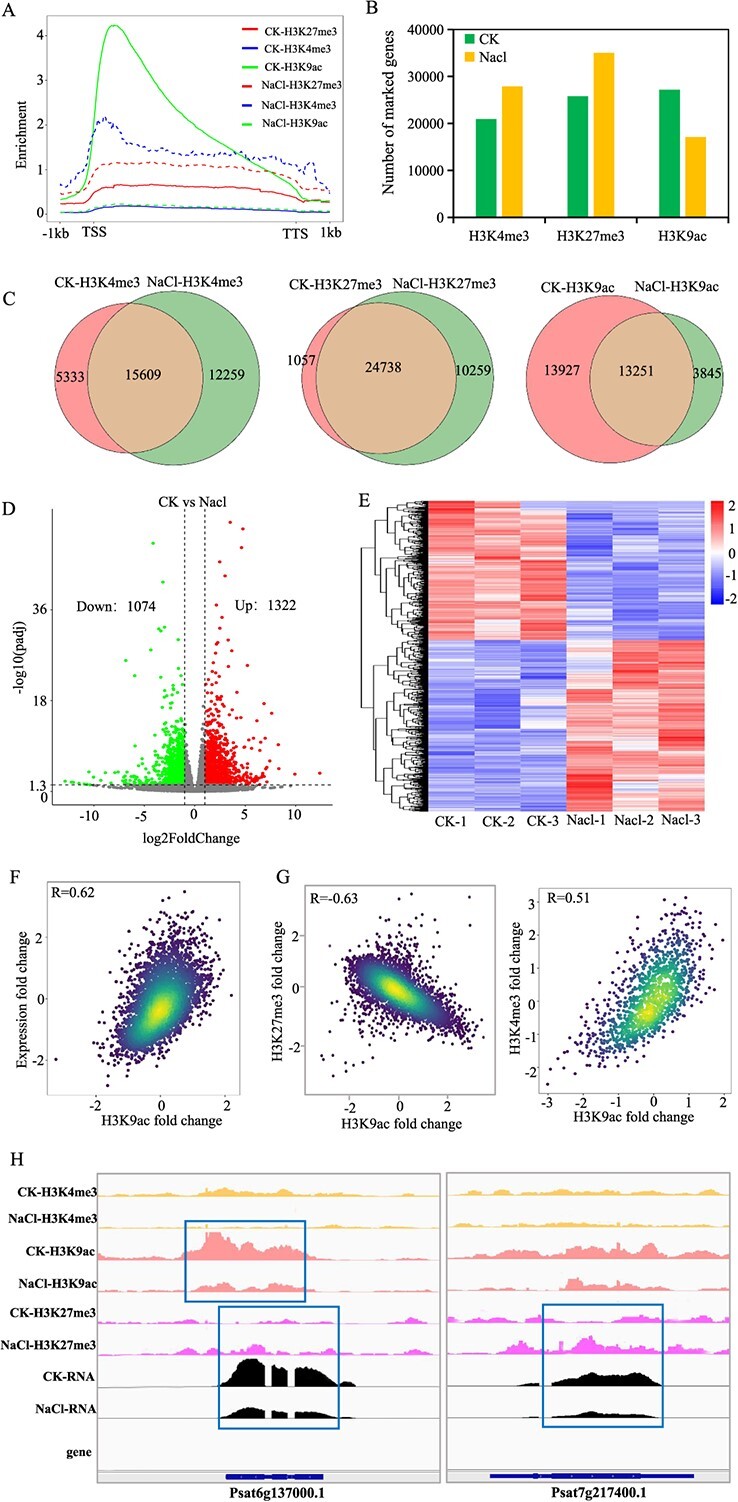
**Relationship between the dynamic changes of single histone modification enrichment and gene expression between CK and NaCl treatment group**. **A.** Levels of histone modifications in genic regions under CK and NaCl treatments. **B.** Number of genes marked by H3K4me3, H3K9ac, or H3K27me3 between CK and NaCl treatment groups. **C.** Changes in the number of histone-modified marked genes between ck and NaCl. **D.** Volcano plot of the differentially expressed genes between CK and NaCl treatment group. Red circles, upregulated genes in NaCl treatment; blue circles, downregulated genes in NaCl treatment. **E.** Cluster analysis of DEGs. Color scale indicates the gene expression level. **F.** Correlations of log2 fold changes in gene expression and histone marks (H3K4me3, H3K9ac, or H3K27me3) between CK and NaCl treatments. Each dot represents the change in expression levels and histone marks of the same gene. **G.** Correlations of log2 fold changes in H3K4me3, H3K9ac, or H3K27me3. **H.** Example from D and E showing H3K4me3, H3K27ac, H3K27me3, and gene expression levels around a specific gene in pea seedlings within a blue box.

Post-salinity exposure in pea, we observed a marked alteration in the overall enrichment of H3K4me3, H3K9ac, and H3K27me3 modifications. Specifically, the enrichment of H3K9ac modification plummeted post-salt treatment, while both H3K4me3 and H3K27me3 modifications exhibited a pronounced surge (Fig. 4A). This observation was further corroborated by western blot analysis (Fig. S10). Upon statistical scrutiny, we discerned that the count of genes influenced by histone modifications underwent significant shifts post-salt exposure. Relative to the control (CK), the NaCl-treated group manifested an addition of 12 259 and a subtraction of 5333 for H3K4me3-marked genes, an addition of 10 259 and a subtraction of 1057 for H3K27me3-marked genes, and an addition of 3845 and a subtraction of 13 927 for H3K9ac-marked genes. The data highlights a significant rise in the quantity of genes exhibiting marks of H3K4me3 and H3K27me3, whereas genes featuring the H3K9ac mark showed a notable decrease (Fig. 4B-C).

To delve deeper into the epigenomic dynamics in salt-treated pea and their potential interplay with gene expression, we firstly employed high-throughput RNA-seq. Each sample yielded ~100 Mb clean reads. Over 95% of the clean reads in each sample were mapped to the reference pea genome sequence (Table S1). The high consistency observed across replicates (Fig. S1B) attests to the reliability of the RNA-seq data, rendering it suitable for subsequent analyses. Differential gene expression (DEGs) between the treated and control groups was discerned by contrasting the FPKM values for each gene, using criteria of |log2(fold change) | ≥ 1 and FDR < 0.05. This analysis identified 2396 DEGs, comprising 1322 upregulated and 1074 downregulated DEGs in the control and NaCl treatment groups, respectively (Fig. 4D). A cluster heat map vividly depicted the similarities between samples and the congruence in gene expression within the same class for a given sample group (Fig. 4E).

To examine the possible involvement of these DEGs under salt stress, we undertook GO and Kyoto Encyclopedia of Genes and Genomes (KEGG) enrichment analyses (Table S3). As depicted in Fig. S11, the DEGs predominantly aligned with GO pathways such as oxidative stress response, sequence-specific DNA binding, abscisic acid binding, L-phenylalanine catabolic process, and defense response. The upregulated DEGs largely mirrored the pathways enriched for all DEGs, whereas the downregulated DEGs were primarily associated with pathways like transmembrane transporter activity, UDP glycosyltransferase activity, ion transport, sequence-specific DNA binding, and monooxygenase activity. Notably, these GO pathways are predominantly stress related. In terms of the KEGG pathway, all DEGs, including those that are upregulated and downregulated, predominantly featured enrichment in pathways like plant–pathogen interactions, plant hormone signaling pathways, and the metabolic pathways for specific defense-related secondary metabolites, including phenylpropanoid and flavonoid biosynthesis (Fig. S11). These findings suggest that pea, akin to other plants, primarily counteracts salt stress through oxidative stress response pathways, hormone signal transduction pathways, and the synthesis of specific defense-related secondary metabolites.

Then we systematically characterized the relationship between the three histone modifications and gene expression in both CK and NaCl treatment groups. Notably, the intensities of H3K4me3 and H3K9ac exhibited a robust positive association with shifts in gene expression between the CK and NaCl-treated seedlings. Conversely, H3K27me3 intensity manifested a negative correlation with gene expression in these groups (Fig. 4F–G, Fig. S12). Furthermore, a mild positive correlation was discerned between the multiplicative changes of H3K4me3 and H3K9ac, while a negative correlation was evident between the multiplicative changes of H3K4me3 and H3K27me3. This aligns with prior findings from diverse plant studies [45–48]. Intriguingly, a pronounced negative correlation was observed between the multiplicative changes of H3K9ac and H3K27me3. For the majority of genes experiencing concurrent changes in H3K27me3 and H3K9ac, these modifications were negatively associated with shifts in gene expression. This is one of the characteristics of H3K9ac and H3K27me3 as bivalent modification. (Fig. 4G–H, Fig. S12).

**Figure 5:**
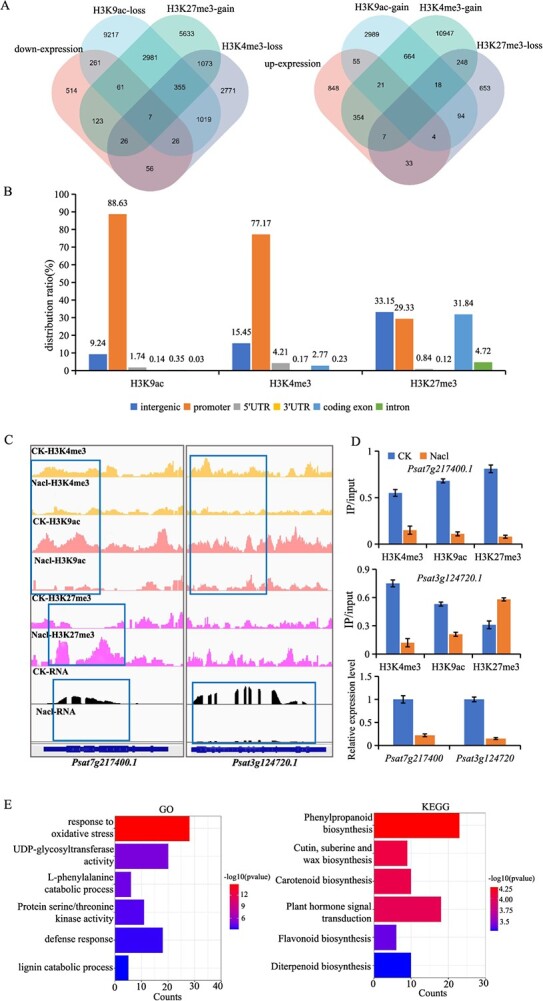
**Characteristics of genes with altered expression caused by histone modification.** A. Venn diagram showing the overlap of down-expression genes and H3K9ac-loss， H3K27me3-gain， H3K4me3-loss genes, the up-expression gene and H3K9ac-gain， H3K27me3-loss， and H3K4me3-gain genes. **B.** Distribution ratio of gene regions corresponding to histone modifications and changes in gene expression levels. **C.** Representative examples of genes marked histone modification between CK and NaCl. **D.** RT-qPCR and ChIP-qPCR test of expression level and histone modification enrichment in B genes. Bars = mean ± SD from three biological repeats. **E.** GO and KEGG enrichment analysis of genes regulated by histone modifications between CK and NaCl treatments, with color scale indicating the significance (−log 10 (*P*-value)) of enriched GO and KEGG terms.

Based on the overall trend of modification changes after salt treatment and the correlation between modification changes and gene expression changes, we investigated the dynamics of genes marked by H3K4me3, H3K9ac, and H3K27me3 modifications and their expression levels in both the control (CK) and treatment groups. The analysis revealed that over half of the downregulated genes (560/1074) and over a third of the upregulated genes (474/1322) were influenced by one or more of the three histone modifications (Fig. 5A). These observations are consistent with previous research conclusions. This suggests that the expression changes of these 1034 genes might be modulated by the three histone modifications. We separately calculated the proportions of H3K9ac, H3K4me3, and H3K27me3 modifications in gene regions for genes associated with H3K9ac-gain/up-expression and H3K9ac-loss/down-expression, H3K4me3-gain/up-expression and H3K4me3-loss/down-expression, as well as H3K27me3-gain/down-expression and H3K27me3-loss/up-expression. Statistical results indicate that the enrichment rate of genes associated with H3K9ac-gain/up-expression and H3K9ac-loss/down-expression on promoters reaches 88.63%, while the enrichment rate of genes linked to H3K4me3-gain/up-expression and H3K4me3-loss/down-expression on promoters reaches 77.17%. In contrast, genes associated with H3K27me3-gain/down-expression and H3K27me3-loss/up-expression are predominantly enriched in intergenic regions, promoters, and coding exons. These findings suggest that the distribution of H3K9ac and H3K4me3 is most closely related to positive regulation of gene expression in promoter regions, whereas H3K27me3 is most prevalent in gene body regions and is most strongly associated with negative regulation of gene expression (Fig. 5B). We arbitrarily selected two of these genes for further analysis and found a correlation between their expression levels and the modification function (Fig. 5C). Concurrently, RT-qPCR and ChIP-qPCR validations were conducted (Fig. 5D). In addition, we also selected 10 genes, respectively, whose expression level changes may be caused by corresponding changes in histone modifications, and conducted ChIP-qPCR and RT-qPCR validation in the CK and treatment groups (Fig. S13).

Subsequent GO and KEGG enrichment analyses on these 1034 genes were performed. GO enrichment pinpointed primary associations with oxidative stress response, UDP glycol transferase activity, L-phenylalanine catalytic process, protein serine/threonine kinase activity, and defense response. KEGG enrichment highlighted their involvement in pathways like phenylpropanoid biosynthesis, cutin, suberin, wax biosynthesis, plant hormone signal transduction, carotenoid biosynthesis, flavonoid biosynthesis, and diterpenoid biosynthesis (Fig. 5E). These findings indicate that genes potentially regulated by histone modification following salt exposure in peas are integral to metabolic pathways.

### Histone modifications coordinate the Phenylpropanoid biosynthesis pathway in response to salt stress

To further elucidate the function of histone modifications in response to salt stress, we undertook LC–MS/MS non-targeted metabolomic analysis of pea seedlings exposed to NaCl, juxtaposed with control group seedlings from the same developmental stage as those used in ChIP-seq and RNA-seq experiments. In the study, 622 metabolites were identified utilizing positive ion mode and 537 utilizing negative ion mode, with each sample analyzed in five biological replicates. The identification of differential metabolites relied on specific parameters: VIP > 1.0, FC > 1.5 or FC < 0.667, and *P*-value <0.05. Relative to the control, salt-treated samples exhibited significant variations in 190 positive ion mode metabolites (139 upregulated and 51 downregulated) and 197 negative ion mode metabolites (158 upregulated and 39 downregulated) (Fig. S14).

**Figure 6:**
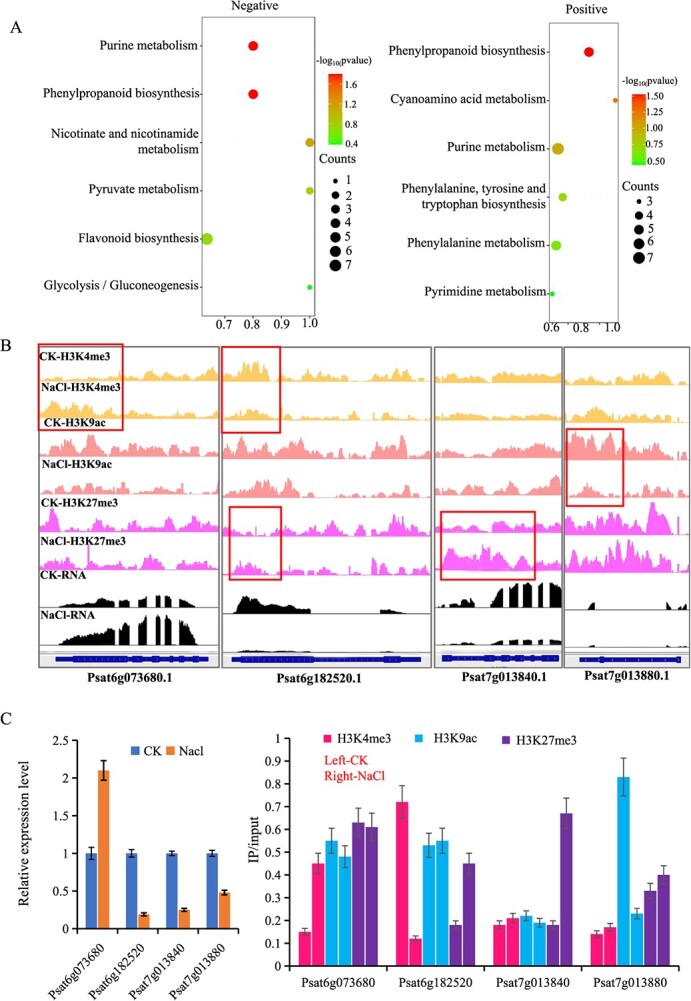
**Genes enriched in the phenylpropanoid biosynthesis pathway are regulated by histone modification. A.** KEGG enrichment of different metabolites gene (DMG) between CK and NaCl. Color scale indicates the significance (−log 10 (*P*-value)) of the enriched KEGG terms. **B.** Representative examples of genes marked histone modification in phenylpropanoid biosynthesis pathway from CK to the treatment group. **C.** RT-qPCR and ChIP-qPCR test of expression level and histone modification enrichment in B genes. Bars = mean ± SD from three biological repeats.

Subsequent pathway annotation of all identified metabolites was executed, followed by KEGG enrichment analysis of all differential metabolites. The most significantly enriched pathways for negative ion mode metabolites included Nicotinate and Nicotinamide Metabolism, Purine Metabolism, Phenylpropanoid Biosynthesis, Pyruvate Metabolism, Flavonoid Biosynthesis, and Glycolysis/Gluconeogenesis. For positive ion mode metabolites, the top pathways were Phenylpropanoid Biosynthesis, Phenylalanine, Cyanoamino Acid Metabolism, Tyrosine and Tryptophan Biosynthesis, Purine Metabolism, Phenylalanine Metabolism, and Pyrimidine Metabolism (Fig. 6A). Notably, the enrichment of genes modulated by histone modifications in response to salt stress (Fig. 5E) prominently highlighted the Phenylpropanoid Biosynthesis pathway, which is highly consistent with our metabolomic data (Fig. 5E, Fig. 6A).

Focusing on this pathway, we categorized genes influenced by histone modifications post-salt exposure. A total of 25 genes exhibited expression shifts in line with histone modifications dynamics (Table S4). Four of these genes were arbitrarily chosen for RT-qPCR and ChIP-qPCR validation, and the results mirrored our sequencing findings (Fig. 6B–C). Taken together, these findings highlight the crucial role of histone modifications in mediating the response to salt stress, primarily through the regulation of gene expression linked to the Phenylpropanoid Biosynthesis pathway in pea.

## Discussion

The pea genome is complex, with an estimated size of ~4.28 Gb, which is four times larger than that of soybeans, six times that of chickpea, seven times that of common beans, and eight times that of mung and adzuki beans. Remarkably, >80% of its genome consists of repetitive sequences [30]. Our histone modification data indicates that H3K4me3, H3K27me3, and H3K9ac are primarily located in the intergenic regions of the pea genome (Fig. 1C). This pattern of distribution significantly differs from other species, such as the Castor Bean, where H3K4me3 and H3K27me3 are mainly concentrated in genic regions, with minimal presence in intergenic areas [50]. Such genic enrichment of these histone marks appears to be a conserved trait in angiosperms [9, 47, 51, 52]. Generally, among the modifications, H3K27me3 exhibits the most pronounced presence in intergenic regions, while H3K9ac has the least. However, our results showed that under natural conditions, H3K4me3 dominated the intergenic distribution at 65.81%, whereas H3K9ac was the least distributed at 45.28%. Post-salt treatment, H3K9ac surged to the forefront with 86.55% distribution in intergenic regions, H3K4me3 receded to 56.82%, and H3K27me3 remained relatively stable, fluctuating between 75.81% and 78.84% (Fig. 1C). These findings suggest a significant presence of histone modifications on the pea’s repetitive sequences. The implications of such modifications on these sequences warrant deeper exploration. Such investigations could expand our understanding of histone modifications, suggesting they not only modulate gene expression but also might influence other genomic elements.

From the perspective of gene enrichment, H3K4me3, H3K27me3, and H3K9ac in peas are significantly enriched in genes, accounting for a large majority of the total gene count, which is also different from other species such as *Arabidopsis* and rice [9, 38, 39, 45, 51]. Specifically, the proportions for these three modifications stood at 46.8%, 57.6%, and 60.7%, respectively. Notably, 15 125 genes exhibited both H3K4me3 and H3K27me3 modifications, representing 33.8% of the total gene count. Meanwhile, 19 900 genes displayed both H3K27me3 and H3K9ac modifications, accounting for 44.5% of the total. Furthermore, 12 441 genes simultaneously bore all three types of modifications, making up 27.8% of the total gene count (Fig. 1A). These results underscore the pivotal role of histone modifications in regulating gene expression in peas, with more than half of the genes being concurrently modulated by multiple modifications. When peas are subjected to salt stress, there is a significant increase in the count of genes enriched with H3K4me3 and H3K27me3, while the count of genes enriched with H3K9ac decreases notably (Fig. 4B). At the whole-genome level (Fig. 4A), a substantial surge in H3K4me3 enrichment is observed, whereas H3K9ac enrichment diminishes significantly, reflecting the observed changes in gene modification enrichment. Interestingly, the genome-wide enrichment of H3K27me3 did not show significant changes, consistent with the findings from the western blot analysis (Fig. S10). This suggests that during salt stress, although the number of genes enriched by H3K27me3 increases, the intensity of this enrichment diminishes. Consequently, as a multitude of genes experience increased enrichment by H3K4me3 post-stress, and with H3K27me3 also on the rise, these genes predominantly exhibit upregulated expression (Fig. S10). This underscores the activating role of H3K4me3 in promoting gene expression. Furthermore, the observed differences in modification intensity further emphasize the complex interplay of histone modifications in regulating gene expression. Conversely, the differences observed between changes in numerous genes and alterations in histone modifications imply that some changes in histone modifications may not directly cause variations in gene expression, highlighting the complexity of histone modification changes that remains to be explored.

Regions containing both repressive and active chromatin modifications are termed bivalent domains [53]. H3K27me3 and H3K4me3 are the earliest proposed bivalent modifications [54]. In animals, H3K4me3 and H3K27me3 are usually co-localized in gene promoter regions, and they coordinate the differentiation of specific cell types [53–55] and the occurrence of cancer through a balance of modification interactions. In plants, several studies have documented the presence of such bivalent modifications (H3K4me3-H3K27me3) during developmental and stress response stages [40, 56–58]. Apart from the aforementioned bivalent modifications, another type of substance called ‘bivalent enhancers’ has been found in both animals and oil-seed rape [45, 47, 59], which is related to cell differentiation and tissue-specific gene expression. In rice, a unique bivalent modification has also been discovered, combining the active mark H3K4me1 with the repressive mark H3K9me2 in CS Copia [60]. The discovery of these bivalent modifications highlights the vital importance of histone modifications in both organismal development and adaptation to environmental variations. These modifications exist in a delicate balance, maintaining the complex equilibrium of gene regulation. We have identified a novel bivalent modification (H3K9ac-H3K27me3) in peas that has not been reported in animals or plants. This bivalent modification primarily occurs in the promoter regions of genes that exhibit specific expression patterns (Fig. 3). These genes are mainly involved in developmental stages such as cell division and short-day cycles, as well as ABA and SA metabolic processes. The roles of these two hormone pathways in plant stress responses have been well-documented, indicating that this novel bivalent modification regulates specific developmental stages and stress responses by controlling gene expression and hormone metabolism. Furthermore, this bivalent modification is significantly enriched in secondary metabolic pathways, such as lignin, apocarotenoid, and terpene metabolism. These metabolites also participate in plant stress responses, and some are major nutritional components of peas as a food source. Our findings reveal a novel bivalent modification with regulatory effects distinct from other bivalent modifications, and the detailed mechanisms of this bivalent modification in regulating gene expression remain to be investigated.

Moreover, following salt stress exposure, most prior studies have primarily focused on changes in histone modifications within stress-responsive genes [12–25]. However, through our integrated analysis using transcriptomics, ChIP-seq, and metabolomics, we discerned that histone modifications can directly target genes associated with the Phenylpropanoid synthesis metabolic pathway. These modifications actively regulate the expression of genes linked to Phenylpropanoid synthesis, subsequently influencing the production of secondary metabolites essential for stress response (Fig. 6). This insight provides a new perspective on the molecular mechanisms through which histone modifications influence stress responses. It suggests a more immediate and direct strategy for organisms to counteract stress conditions.

## Conclusion

In conclusion, a comprehensive characterization of four major histone modifications in pea: H3K4me3, H3K27me3, H3K9ac, and H3K9me2 was present in this study. A comprehensive analysis of four histone modifications (H3K4me3, H3K27me3, H3K9ac, and H3K9me2) and transcriptome in pea was present in this study, and we established a global map of genome-wide regulatory elements, chromatin states, and dynamics based on these major modifications. A new bivalent chromatin state (H3K9ac-H3K27me3) was identified in pea, which is related to growth and development and stress response. Additionally, we elucidated the relationship between histone modification changes and gene expression changes in pea after salt stress. H3K4me3 and H3K9ac are linked to increased gene expression, whereas H3K27me3 is correlated with decreased gene expression. We also unveil that these histone modifications synergistically regulate metabolic genes, influencing metabolite pathways under salt stress conditions.

## Materials and methods

### Plant material

The experiment utilized the dwarf pea variety ‘Zhongwan 11’. This high-yield and superior-quality pea variety is characterized by its broad adaptability, robust stress resistance, abbreviated growth period, and exceptional quality and yield.

Material planting and salt stress treatment

1. **Seeding:** A 32-hole tray measuring 540 × 280 × 110 mm was employed. The tray was lined with vermiculite, and one pea seed was sown in each hole. After sowing, the seeds were covered with additional vermiculite.
2. **Stress Treatment:** A 1.0% (m/v) NaCl solution was prepared using a nutrient solution. A control (CK) was also set up without any NaCl. The seeded tray was immersed in the nutrient solution with the designated salt concentration until thoroughly saturated. Our experimental materials were germinated directly in nutrient solution with added salt. Subsequently, it was covered with a transparent plastic lid and cultivated under specific conditions (25°C, with a light/dark cycle of 16 h/8 h at 4000 l×).

### Sampling and measurement indicators

Twenty days post-sowing, six seedlings exhibiting uniform growth were selected. Their roots were cleaned, and both the SFW and the RFW were recorded. The vitamin C content in the leaves was determined using the 2,6-dichlorophenol indophenol method. The soluble sugar content in the leaves was ascertained using the anthrone method, while the soluble protein content was measured employing the Coomassie brilliant blue method. All remaining seedlings (both aboveground tissues and roots) that displayed consistent growth were chosen and flash-frozen using liquid nitrogen, reserved for subsequent experiments including ChIP-seq, RNA-seq, and metabolome.

### RNA-seq and transcriptome analysis

Total RNA was isolated utilizing the Trizol Reagent (Invitrogen Life Technologies). The RNA was then evaluated and measured in two steps: [1] its purity and concentration were assessed utilizing a NanoDrop spectrophotometer (Thermo Scientific) and a Qubit 4.0 fluorometer; [2] RNA integrity and quantity were measured utilizing the Agilent 2100/4200 system. Three micrograms of RNA were utilized as the starting material for RNA sample preparation. Poly(A+) enrichment and cDNA library construction were performed with Hieff NGS® DNA Selection Beads. Qubit was used for quantification after library construction. Then, the sequencing libraries were run on a DNBSEQ-T7 platform using the PE150 model at 563 sci-service Biosciences Co., Ltd. (Wuhan, China). RNA-seq was conducted with three biological replicates. Quality control of the raw data in FASTQ format was carried out using fastp (v0.21.0) [61] software, which filtered the sequences to yield high-quality clean data. The clean reads were aligned to the reference genome utilizing HISAT2 (v2.1.0) [62]. Read counts were determined with StringTie (v2.1.5) (https://ccb.jhu.edu/software/stringtie/), and fragments per kilobase of exon model per million mapped fragments (FPKM) were utilized to standardize the expression. DEGs analyzed by DESeq2 (v1.34.1) [63] were selected among all genes with a filter of |log2FoldChange| > 1, significant *P*adj ≤ 0.05. Afterwards, the selected DEGs were compared to the GO and KEGG database for functional annotation (*P*adj ≤ 0.05), and their functions and metabolic pathways will be analyzed and predicted.

### Metabolome profiling and statistical analysis

Untargeted metabolites were analyzed utilizing a Vanquish UHPLC system coupled with an Orbitrap Q ExactiveTM HF mass spectrometer (Thermo Fisher, Germany). Each sample, replicated five times, consisted of 100 mg of tissue ground in liquid nitrogen, resuspended in 80% methanol, vortexed, chilled on ice for 5 min, and centrifuged at 15 000 g at 4°C for 20 min. A fraction of the supernatant was diluted to a final methanol concentration of 53% with LC–MS-grade water. A Hypesil Gold column (Thermo Fisher, Germany) was used in this study. Analyses were carried out in both positive and negative ionization modes. Raw data were processed with Compound Discoverer 3.1 (CD3.1, Thermo Fisher) for peak alignment, detection, and quantification, with a mass tolerance set at 5 ppm. Peak intensities were normalized to the total spectral intensity, aiding in molecular formula prediction by considering additive ions, molecular ion peaks, and fragment ions. PCA and Spearman Correlation analysis were used to assess sample repeatability and quality control. The metabolites identified were categorized and linked to metabolic pathways using databases such as KEGG, HMDB, and LIPIDMaps. Group differences were quantified and statistically analyzed, with each compound’s significance determined by a *t*-test. Metabolites identified as differential displayed a VIP > 1, *P*-value <0.05, and fold change (FC) ≥ 2 or FC ≤ 0.5. A hypergeometric distribution test was conducted to determine significant pathway enrichments among the differentiated metabolites.

### Western blot analysis

For the western blot analysis, histone-enriched fractions were isolated from both CK and NaCl-treated seedlings utilizing the EpiQuik Total Histone Extraction Kit (OP-0006-100). The extracted histones were dissolved in Laemmli sample buffer (62.5 mm Tris–HCl, pH 6.8, 2% SDS, 25% glycerol, 0.01% bromophenol blue, and 10% β-mercaptoethanol). The histone samples were subjected to a 15% SDS-PAGE for separation and subsequently transferred onto a PVDF membrane. The membrane was blocked with 2% bovine serum albumin in phosphate-buffered saline (pH 7.5) and incubated with primary antibodies such as anti-H3 (ab1791, Abcam), H3K4me3 (07–473; Millipore), H3K27me3 (a2363; ABclonal), and H3K9ac (07–352, Millipore) at a dilution of 1:5000 for 1.5 h at room temperature. After washing four times with PBST (15 min each), the membrane was incubated with a secondary antibody diluted at 1:10 000 for 1 h, and then rinsed three times with PBST (15 min each). Visualization was performed utilizing the ECL Enhanced Kit (RM00021, ABclonal). The anti-H3 is used to adjust the amount of histone loaded.

### Chromatin-immunoprecipitation and Re-ChIP

Prior to initiating ChIP experiments, the efficacy of antibodies H3K4me3 (07–473; Millipore), H3K27me3 (a2363; ABclonal), H3K9ac (07–352, Millipore), and H3K9me2 (AB1220, abcam) was validated using the western blot technique. The ChIP experiments were executed following the protocol delineated by Saleh et al [64] and Desvoyes et al [65]. Briefly, ~2 g of pea seedlings were cross-linked with 1% formaldehyde under vacuum for 15 min. The cross-linking was terminated with the addition of 0.125 M glycine for 5 min. Subsequently, the samples were washed, dried, and finely ground into a powder in liquid nitrogen. The chromatin was then extracted, sonicated into fragments smaller than 500 bp, and immunoprecipitated utilizing the validated antibodies. The immunocomplexes were cleansed, eluted, and the cross-links reversed, followed by DNA extraction utilizing phenol-chloroform, purification, and resuspension in distilled water.

Sequential chromatin immunoprecipitation (SeqChIP), or Re-ChIP, involves subjecting formaldehyde-cross-linked protein-DNA complexes from living cells to two consecutive immunoprecipitations using different antibodies. The initial ChIP procedure involved tissue fixation, chromatin sonication, and immunoprecipitation with the primary antibody, which was attached to the beads utilizing disuccinimidyl suberate (DSS, Pierce, 21 555). The protein-DNA complexes were released from the beads by treating them with ChIP elution buffer (50 mM Tris–HCl pH 7.5, 10 mM EDTA, 1% SDS) at 65°C for 15 min after washing. The eluted chromatin was then subjected to a second round of immunoprecipitation using a secondary antibody, followed by elution and DNA purification as in the primary ChIP.

A parallel sample, processed without any antibody, served as the input control. The DNA obtained from both ChIP and Re-ChIP, as well as the input DNA, was reserved for subsequent analyses, including ChIP-seq or RT-qPCR. The primers employed in these analyses are presented in Table S5.

### ChIP-seq and data analysis

ChIP-derived DNA was employed to craft sequencing libraries, adhering to the guidelines of the Illumina TruSeq ChIP Sample Prep Set A. Both library construction and sequencing were undertaken by Novogene (Tianjin, China), with sequencing performed on the Illumina HiSeq-2000 platform.

Inferior reads were removed utilizing the Trimmomatic software (version 0.35) (http://www.usadellab.org/cms/uploads/supplementary/Trimmomatic/Trimmomatic-Src-0.35.zip). The refined data were then aligned to *P. sativum* L reference genome (https://www.peagdb.com/index/) utilizing BWA 39, setting parameters to allow fewer than two mismatches. Reads that had a MAPQ score <30 and redundant PCR were excluded utilizing SAMTools to enhance data quality. To identify enriched regions and call peaks, Model-Based Analysis of ChIP-Seq (MACS) software (version 2.1.4) [66] was utilized, analyzing IP sample reads relative to the input control. For narrow peaks (H3K9ac and H3K4me3) and broad peaks (H3K27me3 and H3K9me2), parameters (macs2 callpeak –t < input file > − c < control file > − f BAM –n < output peak file > − g8.6e8) and (macs2 callpeak –t < input file > − c < control file > − f BAM –n < output peak file > − g 8.6e8 –broad) were used. The Wig files, generated by MACS, facilitated data visualization through IGV (version 2.8.0.01) [67]. Lastly, the DeepTools 3.3.0 [68] software was harnessed to produce heat maps representing different histone marks. The same methodologies were applied for Re-ChIP-seq data analysis, albeit with modified standards (macs2 callpeak–t < input file > − c < control file > -B –broad–broad-cutoff 0.01 -q 0.01 -g 8.6e8 –fe-cutoff 4.0 –nomode) to enhance peak calling.

### Gene ontology and KEGG enrichment analysis

The enrichment analysis of GO and KEGG pathways for DEGs, histone modification genes, and genes regulated by modifications was performed utilizing AgriGOv2 (http://systemsbiology.cau.edu.cn/agriGOv2/) [69] and KOBAS software [70].

### Analysis of chromatin states

CS analysis was conducted utilizing chromHMM (1.21) [47]. To annotate CS, we employed the ‘BinarizeBam’ and ‘LearnModel’ commands, maintaining their default parameters. The model training tested various configurations, with the number of CS varying between 8 and 20. A 13-state model was ultimately selected due to its comprehensive coverage of all pertinent CS data. To assess dynamic changes in CS, 200-bp bins labeled with CS were considered dynamic if their states differed across samples. The variability score for histone modifications was calculated by determining the one-minus-Jaccard index utilizing bedtools (v2.31.0) (https://github.com/arq5x/bedtools2/releases/). Previous studies have similarly employed CS models on data from rice [60], rapeseed [45], *Arabidopsis* [71], and wheat [72].

### RT-qPCR

Total RNA from CK and NaCl treatment group materials were extracted using the TRIzol reagent (Invitrogen). From this, total RNA (1 μg) were subjected to reverse transcription to yield cDNA, employing ABScript II cDNA First-Strand Synthesis Kit (RK20400, ABclonal). The synthesized first-strand cDNA served as the template for subsequent RT-qPCR analyses.

RT-qPCR was executed on an ABI 7900 system. The amplification conditions were set as an initial denaturation at 95°C for 15 s, followed by 42 cycles of 95°C for 5 s, and 60°C for 40 s. The dissociation curve analysis was conducted with the following steps: 95°C for 15 s, 60°C for 20 s, and a final 95°C for 15 s. Data collection and analysis were carried out utilizing the ABI 7900 system. Gene expression levels were quantified utilizing using the 2 ^-△△CT^ method [73]. For normalization purposes, GH720838 (transcription factor IIA) was employed as the reference gene [74]. The specific primers utilized for RT-qPCR are detailed in Table S5.

## Supplementary Material

Web_Material_uhae259

## Data Availability

All raw sequencing data generated in this project, including RNA-seq and Chip-seq, were deposited at GSA (https://www.ngdc.cncb.ac.cn/) under BioProject accession number PRJCA020204.
