# Supplementary material for: Genome-wide mapping of main histone modifications and coordination regulation of metabolic genes under salt stress in pea (Pisum sativum L)
Source: Hortic Res. 2024 Sep 16;11(12):uhae259. doi: 10.1093/hr/uhae259 (PMC11630261; doi:10.1093/hr/uhae259)
Supplement: Web_Material_uhae259 [file web_material_uhae259.zip › supplemental figures.pdf]

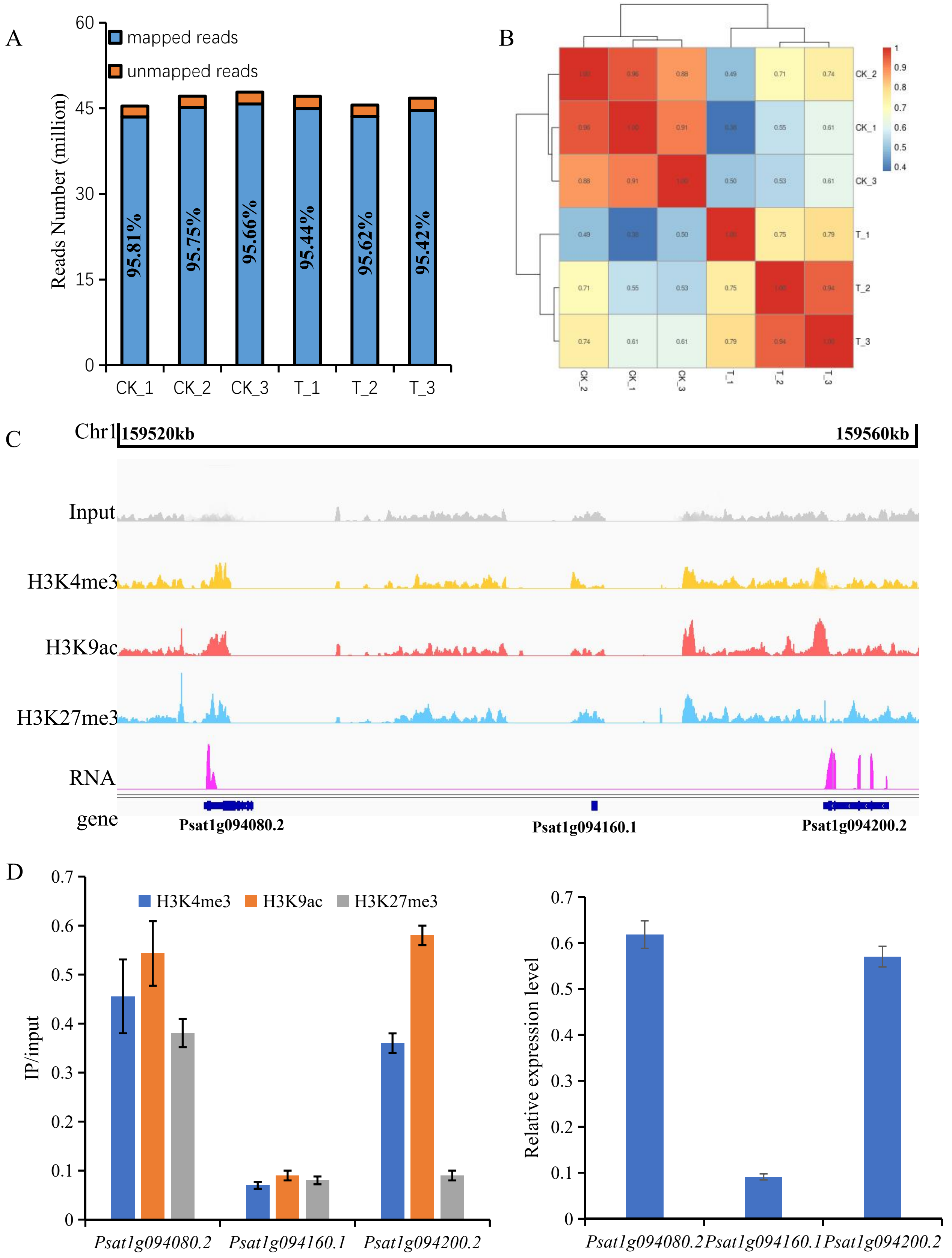


**FigureS1. ChIP-seq and RNA-seq data quality assessment.**

A. The mapping rate of RNA-SEQ;

B. The correlation between each biological replicates of control and salt treated for RNA-SEQ ;

C. Snapshots of ChIP-seq data (scaffold: Chr1, 159,520 kb - 159,560 kb);

D. Validation of some random genes' enrichment and transcriptional levels with quantitative RT- PCR . Bars are means ± SD from three biological replicates.

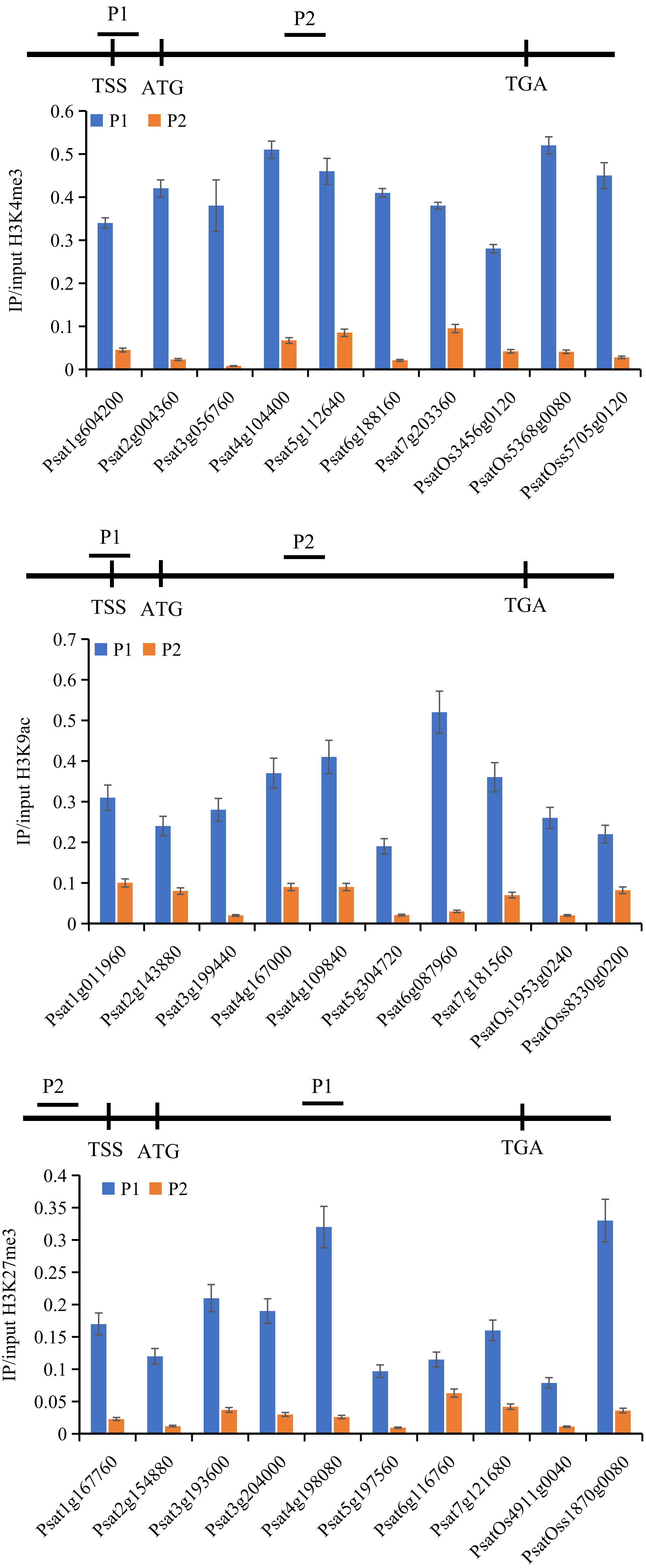


**FigureS2. Verify the enrichment of some selected genes using ChIP-qPCR to validate the accuracy of ChIP-seq data.** Bars are means ± SD from three biological replicates. P1-P2 represent the primer pairs used in the qPCR.

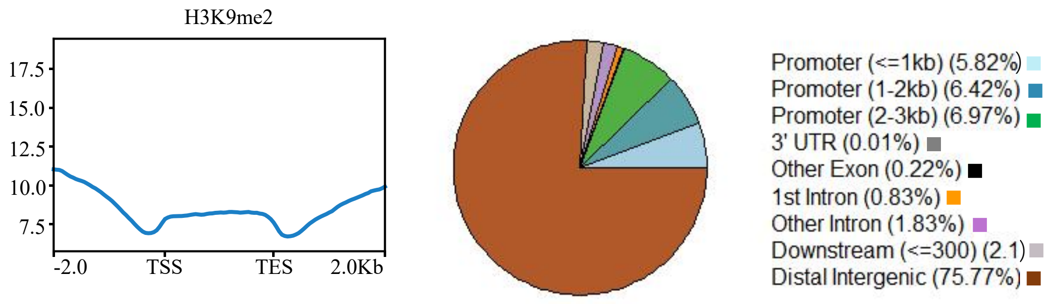


**FigureS3. Characterization of H3K9me2 in Pea**.

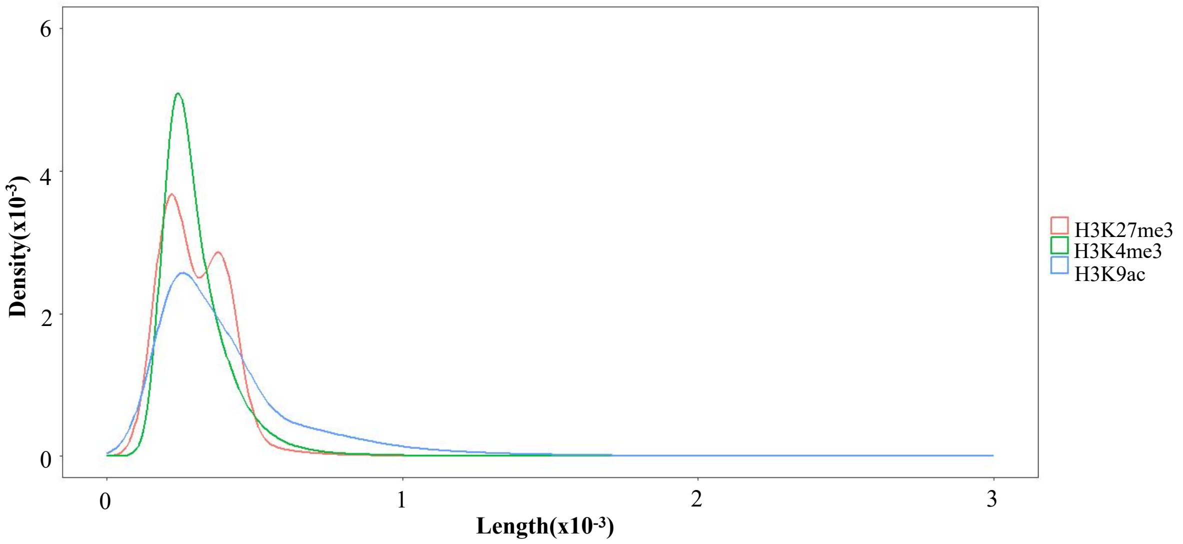


**FigureS4. Length of histone marked regions in pea.**
The genome-wide distribution of total peaks called from the three histone marks by using ChIP-seq according to their length in sorghum.

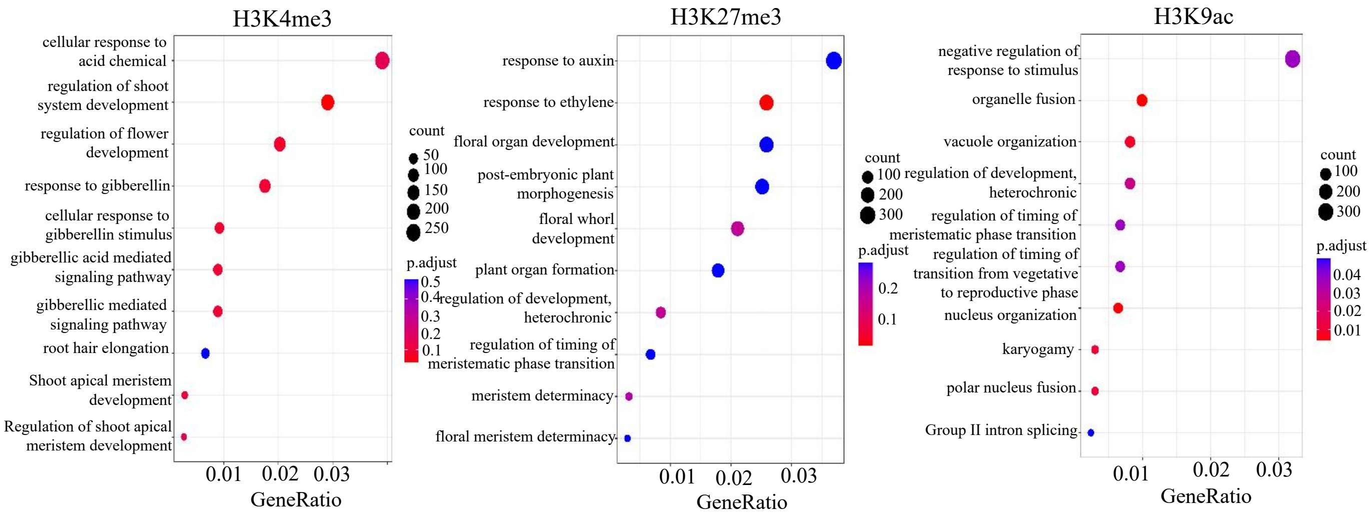


**FigureS5. GO enrichment of H3K4me3,H3K27me3 and H3K9ac marked genes.** Color scale indicates the significance (p-adjust)) of the enriched GO terms.

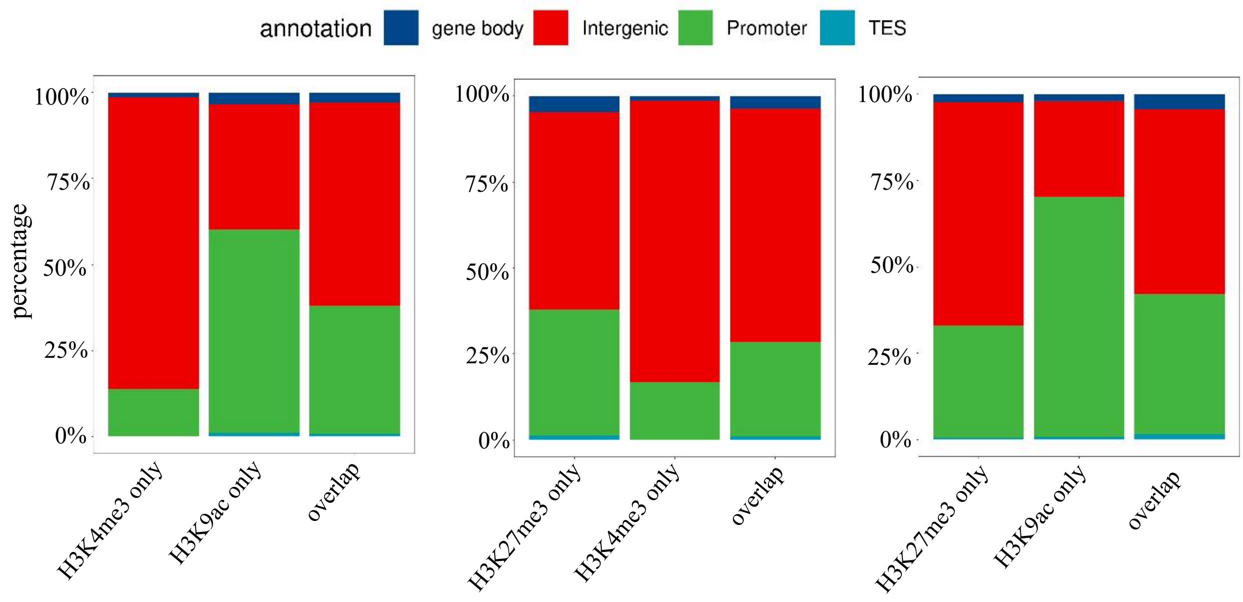


**FigureS6. Characteristics of the genes marked by two or three histone modifications.**

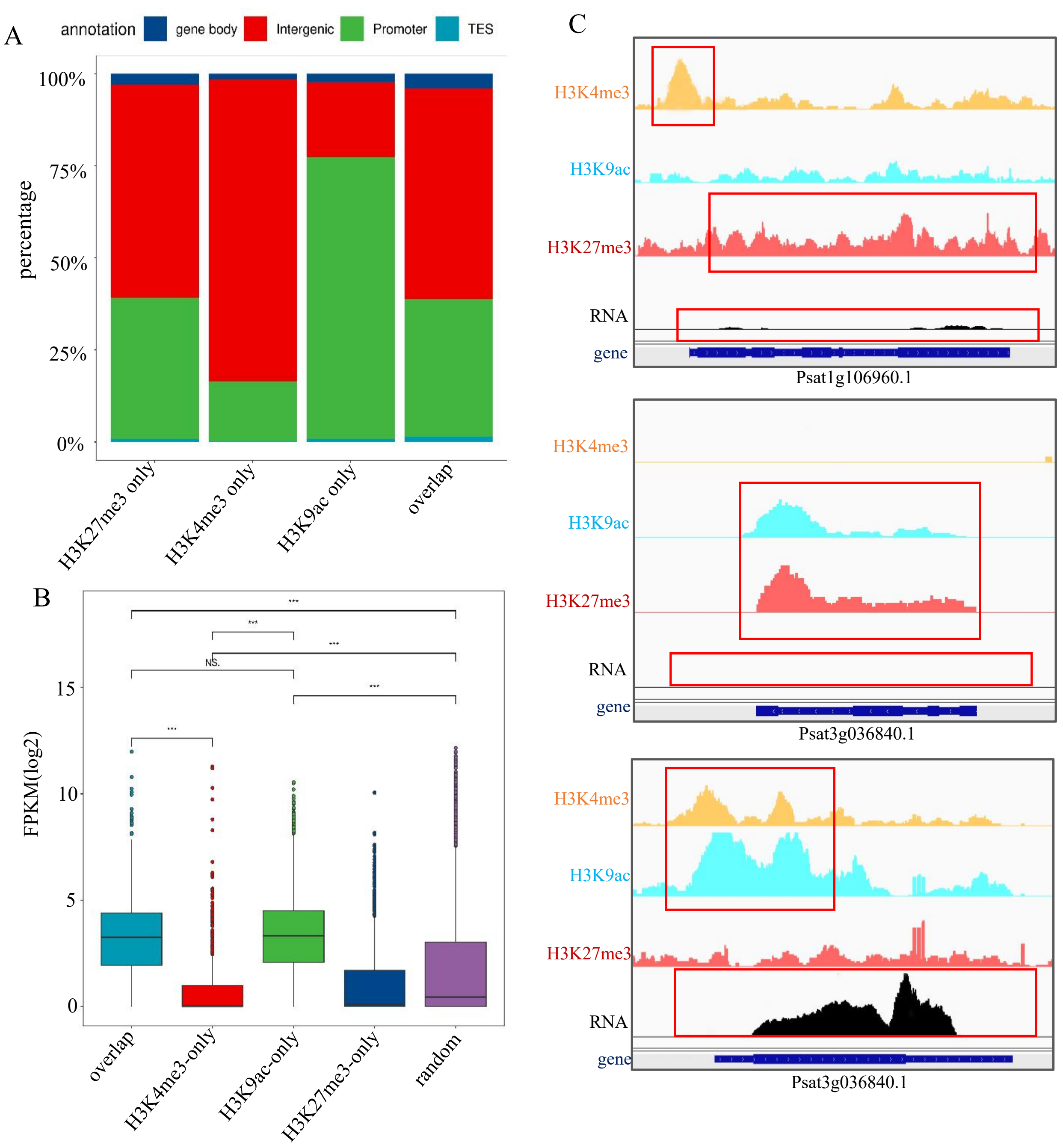


**FigureS7. Characteristics of genes marked by multiple histone modification.**

**A.** Distribution of regions modified by H3K9ac, H3K4me3, and H3K27me3 in the pea genome; ''overlap'' refers to the regions that are overlap marked by all three modifications.

**B.** Expression breadth of genes in the indicated categories. The Wilcoxon rank-sum test was used for testing the statistical significance of differences; 'random' refers to 10 000 randomly selected genes. Significant differences are indicated by asterisks (Wilcox. Test, $p<0.001$).

**C.** Genome browser screenshot showing genes marked by H3K4me3 and H3K27me3 , H3K4me3 and H3K9ac, H3K9ac and H3K27me3 , respectively. The H3K4me3, H3K9ac, H3K27me3 and gene expression levels in pea seedling around a gene within the red box.

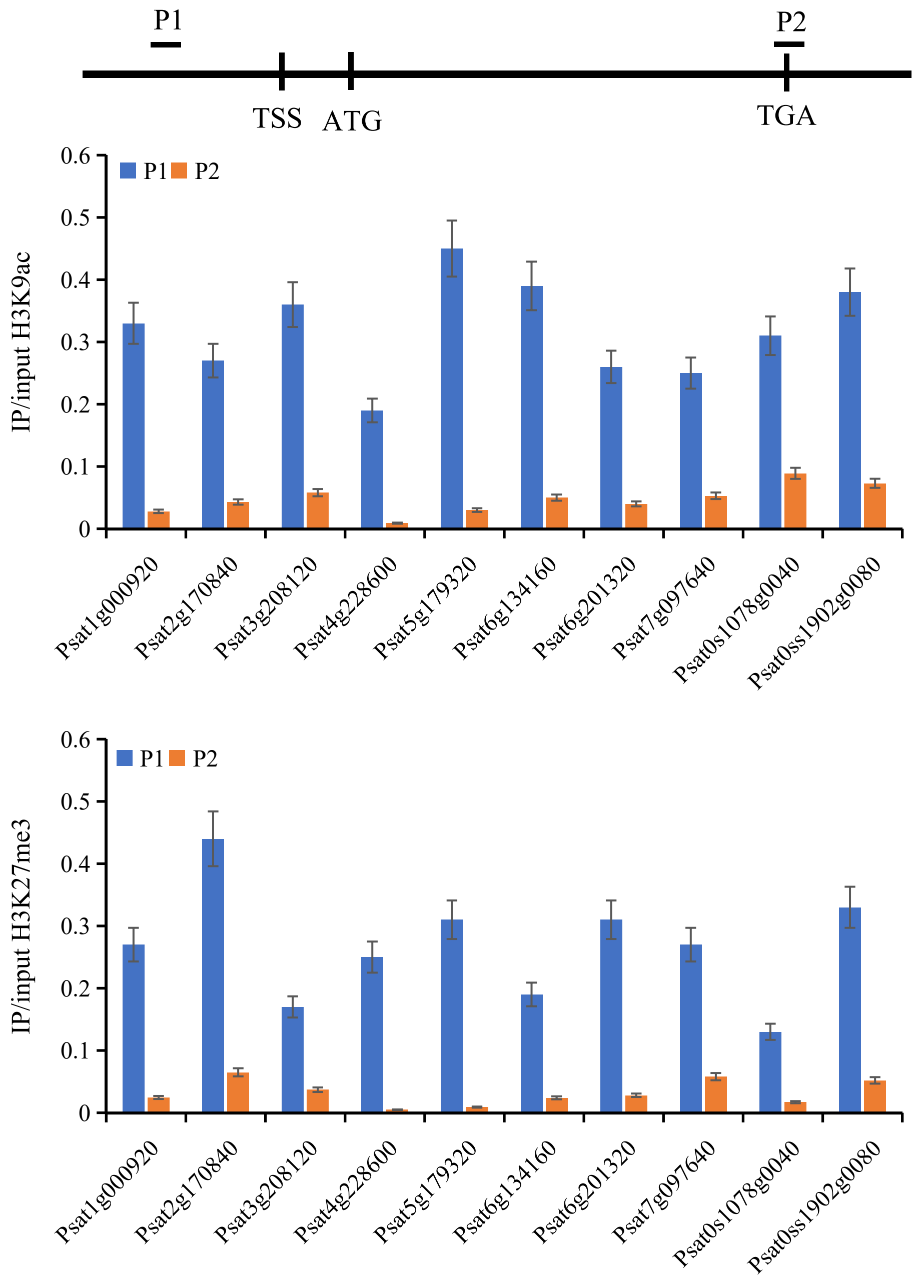


**FigureS8. Verify the enrichment of some selected genes using ChIP-qPCR to validate the accuracy of Re-ChIP-seq data.** Bars are means ± SD from three biological replicates. P1-P2 represent the primer pairs used in the qPCR.

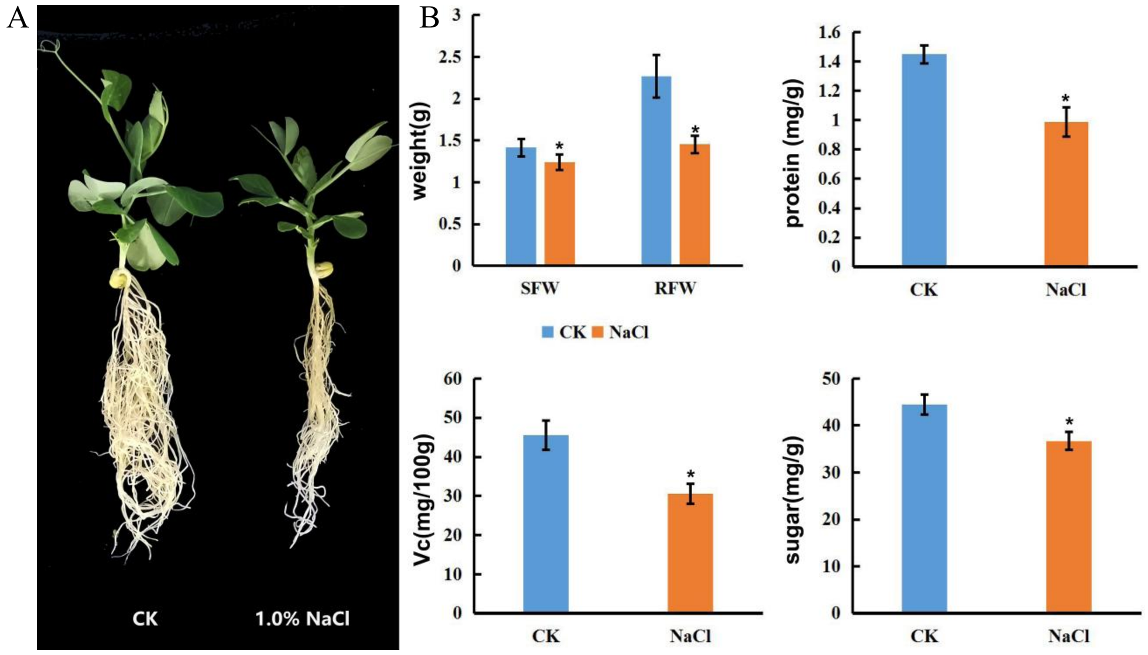


**Figure S9. Growth status and related indicators of pea seedlings under salt stress.**

**A.** Phenotypic changes in whole plants of pea.

**B.** Changes in weight, protein, Vc and sugar between treated plants and their controls (mean±SE), Significant differences are indicated by asterisks (Fisher's tests, P-value <0.05).

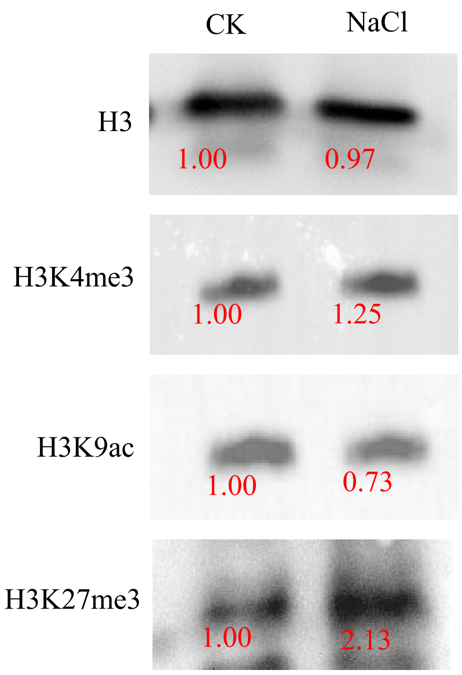


**FigureS10. The level of H3K4me3,H3K27me3 and H3K9ac detected by Western Blot between CK and NaCl in pea seedling.**

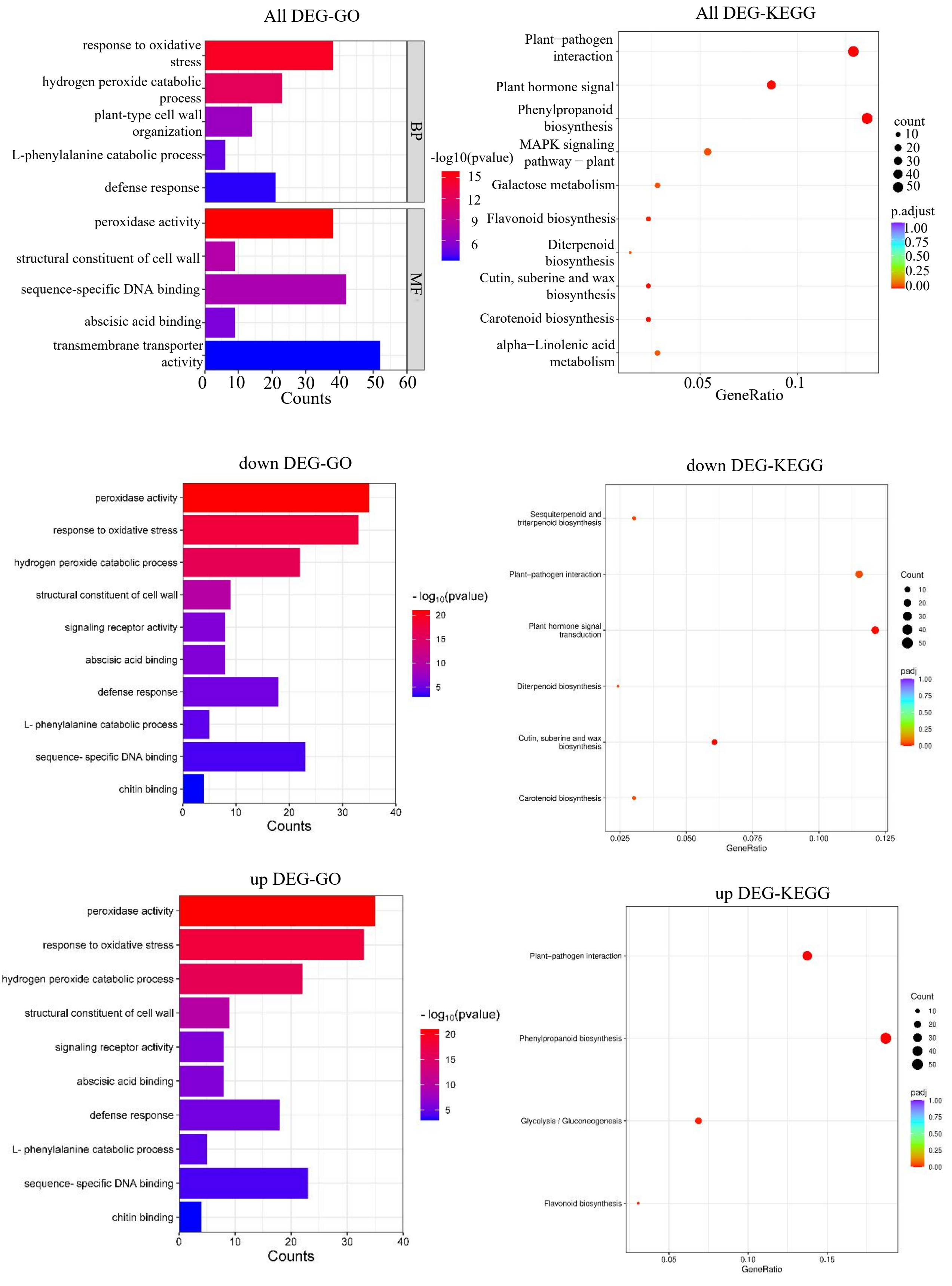


**FigureS11. GO and KEGG analysis of the differentially expressed genes between CK and NaCl treatment group.**

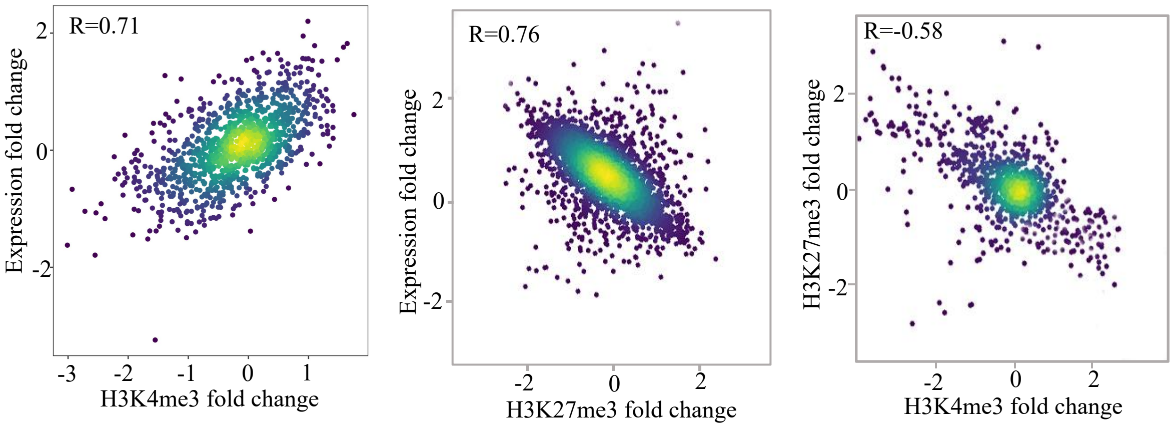


**FigureS12. Correlations of log2 fold changes in gene expression, H3K4me3, and H3K27me3 between CK and NaCl.** Color scale indicates the fold changes in gene expression. Each dot represents the degree of change between expression levels and three types of histone marks of the same gene.

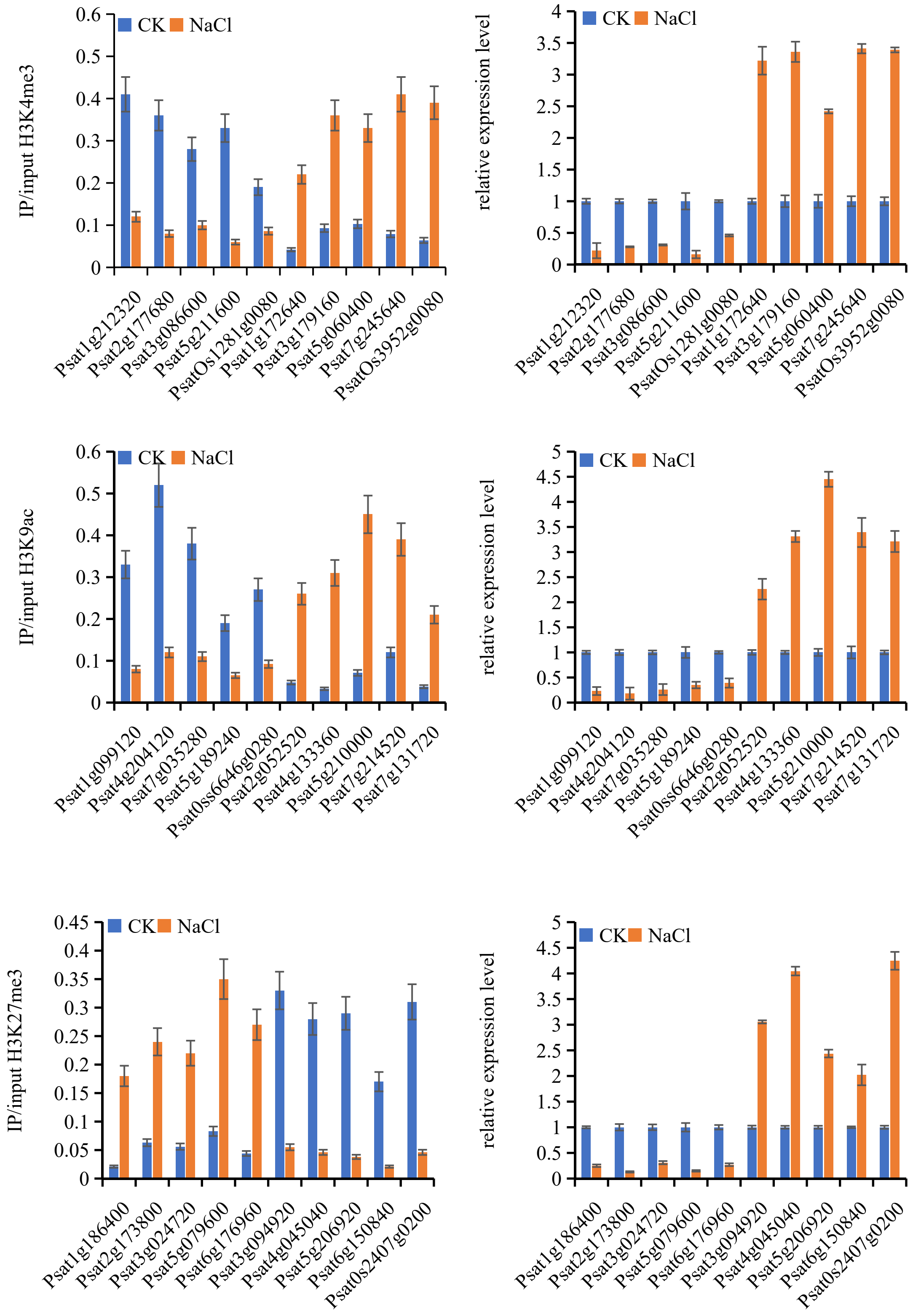


**FigureS13. Verify the enrichment of some selected genes whose expression level changes may be due to corresponding changes in histone modifications using ChIP-qPCR and RT-qPCR verification in both the CK and treatment groups.** Bars are means ± SD from three biological replicates.

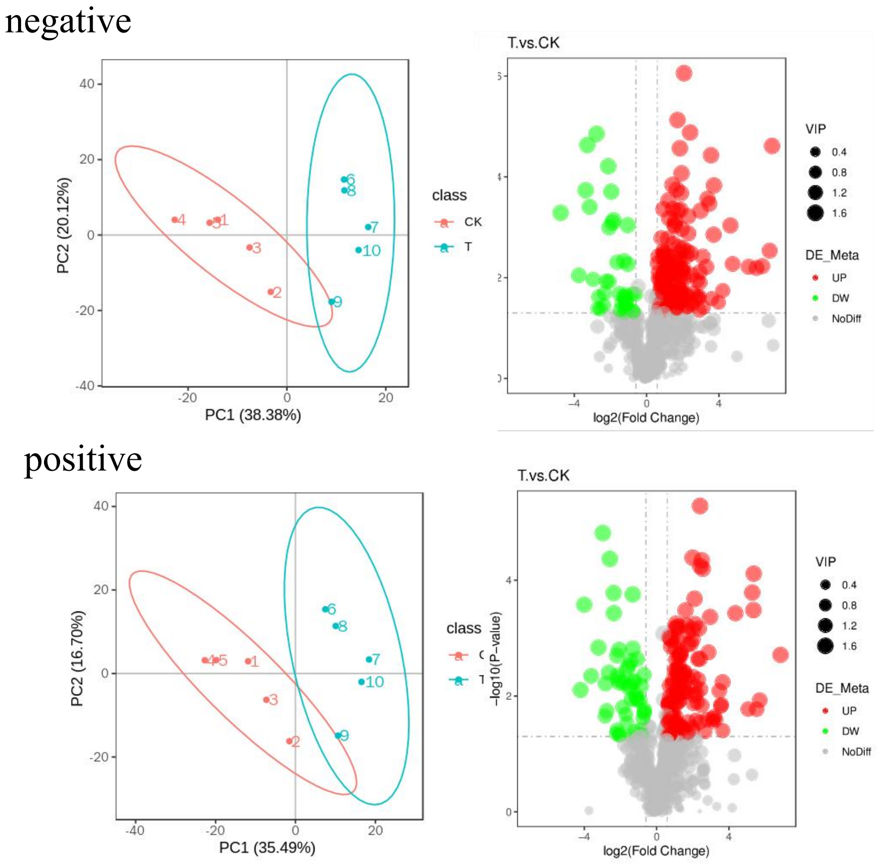


**FigureS14. PCA and different metabolites gene (DMG) in metabolome between CK and NaCl treat group of pea seedling.**
